# Improvement of antibody functionality by structure-guided paratope engraftment

**DOI:** 10.1038/s41467-019-08658-4

**Published:** 2019-02-13

**Authors:** Qingbo Liu, Yen-Ting Lai, Peng Zhang, Mark K. Louder, Amarendra Pegu, Reda Rawi, Mangaiarkarasi Asokan, Xuejun Chen, Chen-Hsiang Shen, Gwo-Yu Chuang, Eun Sung Yang, Huiyi Miao, Yuge Wang, Anthony S. Fauci, Peter D. Kwong, John R. Mascola, Paolo Lusso

**Affiliations:** 1 Laboratory of Immunoregulation, National Institute of Allergy and Infectious Diseases, NIH, Bethesda, MD 20892 USA; 2 Vaccine Research Center, National Institute of Allergy and Infectious Diseases, NIH, Bethesda, MD 20892 USA

## Abstract

Broadly neutralizing antibodies (bNAbs) represent a promising alternative to antiretroviral drugs for HIV-1 prevention and treatment. Selected antibodies to the CD4-binding site bolster envelope trimer binding via quaternary contacts. Here, we rationally engraft a new paratope, i.e., the extended heavy-chain framework region 3 (FR3) loop of VRC03, which mediates quaternary interaction, onto several potent bNAbs, enabling them to reach an adjacent gp120 protomer. The interactive quaternary surface is delineated by solving the crystal structure of two FR3 loop-chimeric antibodies. Chimerization enhances the neutralizing activity of several potent bNAbs against a majority of global HIV-1 strains. Compared to unmodified antibodies, chimeric antibodies display lower autoreactivity and prolonged in vivo half-life in huFcRn mice and rhesus macaques. Thus, paratope engraftment may be used to expand the epitope repertory of natural antibodies, improving their functionality for disease prevention and treatment.

## Abstract (web summary)

Quaternary contacts mediated by an extended heavy-chain framework region 3 (FR3) have been shown to improve binding to HIV envelope and virus neutralization for a few antibodies. Here, Liu et al. engraft such an FR3 loop onto several potent broadly neutralizing antibodies, resulting in improved neutralization activity and pharmacokinetics.

## Introduction

The native HIV-1 envelope (Env) spike is a heavily glycosylated trimer of gp120–gp41 heterodimers, which mediates viral attachment and entry^1^. As the main antigenic structure presented on the virion surface, Env is the sole target of HIV-1 neutralizing antibodies^2^. However, owing predominantly to its inherent flexibility and extensive glycan shield, Env is not efficient at inducing broadly neutralizing antibodies (bNAbs), as inferred by the infrequent and delayed appearance of bNAbs in individuals infected with HIV-1^3^. Accordingly, in spite of major efforts over the past 2 decades, Env-based vaccines have not been successful in eliciting the production of bNAbs^4,5^. Nonetheless, multiple bNAbs directed against major supersites of HIV-1 vulnerability have been isolated from infected individuals, including antibodies to three sites in gp120, i.e., the CD4-binding site (CD4-BS), a glycan-dependent V1V2-loop region at the trimer apex, and a glycan-dependent region at the base of the V3 loop; to a region at the gp120–gp41 interface which includes the fusion peptide; and to the membrane-proximal external region of gp41^6–12^. Among the gp120 supersites, the CD4-BS is the most conserved and less protected by the glycan shield, making it a primary antigenic target for vaccine development.

We recently reported that the functional CD4-BS in the HIV-1 Env trimer has a quaternary nature and identified a second CD4-binding site, designated CD4-BS2, in the inner domain of a neighboring gp120 protomer^13^. We also found that selected anti-CD4-BS antibodies, such as VRC03 and VRC06, mimic the quaternary binding mode of CD4, establishing contacts with two adjacent gp120 protomers^13^. VRC03 and VRC06 belong to the VRC01 class of anti-CD4-BS antibodies, which includes VRC07, N6 and 3BNC117 among others; all these antibodies originate from the same germline heavy-chain gene, VH1–2^14,15^. However, unlike other members of this class, VRC03 and VRC06 contain an extended framework region 3 (FR3) loop in their heavy chain^7^, which was predicted by docking to mediate quaternary contact^13,16^. The heavy-chain FR3 region contains a hypervariable region 4 (HV4)-like domain that may participate in antigen binding and has been reported to be a site for functionally relevant amino acid insertions^17^. A recently published structure of VRC03 in complex with a soluble Env trimer confirmed that the FR3 loop of this antibody interacts with an adjacent gp120 protomer^18^. In contrast, some of the most potent bNAbs, such as VRC01, VRC07, and N6^19–22^, appear to interact with a single gp120 protomer, and indeed were insensitive to mutations in CD4-BS2^13^.

Since the establishment of quaternary contact appears to bolster the interaction of both CD4 and selected antibodies with the Env trimer^9,13,23,24^, we hypothesize that enabling bNAbs specific for a single gp120 protomer to reach the adjacent protomer would result in an increased binding affinity and neutralization potency. To validate this assumption, we engraft the extended FR3 loop of VRC03 onto different CD4-supersite bNAbs, obtaining chimeric antibodies that show improved antiviral activity and pharmacokinetics. These results illustrate a strategy to enhance the biological properties of natural antibodies by transplanting new epitope specificities.

## Results

### Selected anti-HIV-1 antibodies make FR3-loop-mediated quaternary contact

**Fig. 1:**
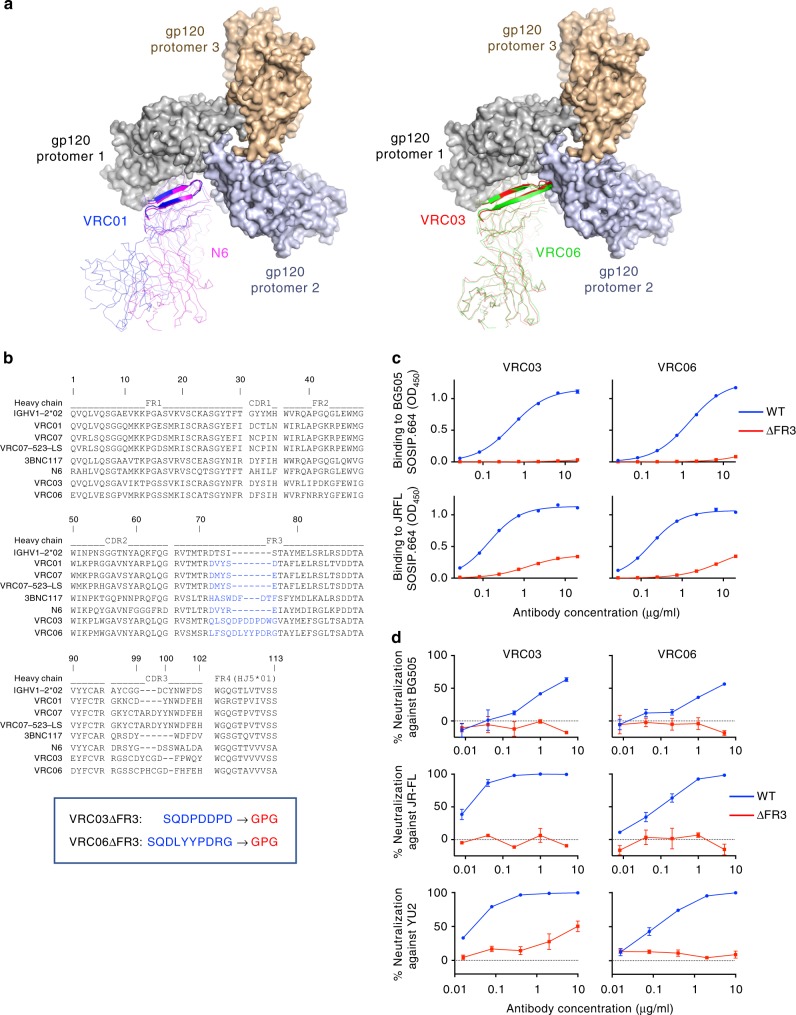
Quaternary interaction of selected antibodies with the HIV-1 Env trimer. **a** Structure alignment shows that certain antibodies like VRC03 (red) and VRC06 (green), unlike other VRC01-class bNAbs like N6 (magenta) and VRC01 (blue), possess an extended heavy chain framework region 3 (FR3) loop which reaches across the interprotomer groove to interact with a neighboring gp120 protomer (protomer 2) in the HIV-1 envelope trimer. **b** Heavy-chain sequence alignment showing the uniquely extended FR3 loops of VRC03 and VRC06 in comparison to those of other anti-CD4 supersite antibodies. Two loop-deletion variants of these two antibodies, VRC03ΔFR3 and VRC06ΔFR3, were designed (indicated at the bottom) by replacing the FR3 loop with a short GPG linker. **c** Decreased binding of FR3-loop-deletion mutants VRC03ΔFR3 and VRC06ΔFR3 to two soluble HIV-1 gp140 trimers (BG505 and JR-FL SOSIP.664), as tested by ELISA. Data represent the mean value with standard deviation (SD) from two replicate wells from a representative experiment out of three performed which yielded similar results. **d** Loss of neutralizing capacity of the FR3-loop-deletion mutants VRC03ΔFR3 and VRC06ΔFR3 against different HIV-1 strains, as tested by TZM-bl assays. Data represent the mean (±SD) from two replicate wells

By docking and mutagenesis studies, we previously demonstrated that certain antibodies to the CD4 supersite, such as VRC03 and VRC06, establish functionally relevant quaternary interaction with two neighboring gp120 protomers in the HIV-1 Env trimer, while others appear to make contact with a single gp120 protomer (Fig. 1a)^13^. Sequence alignment shows that the heavy chains of VRC03 and VRC06 contain an extended acidic FR3 loop (+7 aa), compared to the FR3 loop present in all the other anti-CD4-supersite antibodies, with the only exception of 3BNC117 which contains four additional residues (Fig. 1b). An extension of the CDR1 loop is instead present in VRC-CH31^25^, another antibody that was partially sensitive to mutations in CD4-BS2^13^ (Supplementary Fig. 1). To investigate the functional role of the FR3 loops in VRC03 and VRC06, we generated two deletion mutants, VRC03ΔFR3 and VRC06ΔFR3, in which the FR3 loop was replaced by a tripeptide linker, GPG. The two deletion mutants were efficiently expressed in HEK293 free-style (FS) cells and showed a seemingly correct folding after affinity purification (Supplementary Fig. 2); however, binding to the BG505 (clade-A) and JR-FL (clade-B) SOSIP.664 trimers in ELISA was drastically reduced (Fig. 1c). Furthermore, their neutralizing capacity was nearly abrogated, as shown in TZM-bl cells against pseudoviruses from three different HIV-1 strains (BG505, JR-FL, and YU2) (Fig. 1d). These results demonstrate that the long FR3 loops that mediate quaternary contact play an essential role in the functional interaction of VRC03 and VRC06 with the native HIV-1 Env trimer.

### FR3-loop engraftment enhances the potency of several HIV-1 bNAbs

**Fig. 2:**
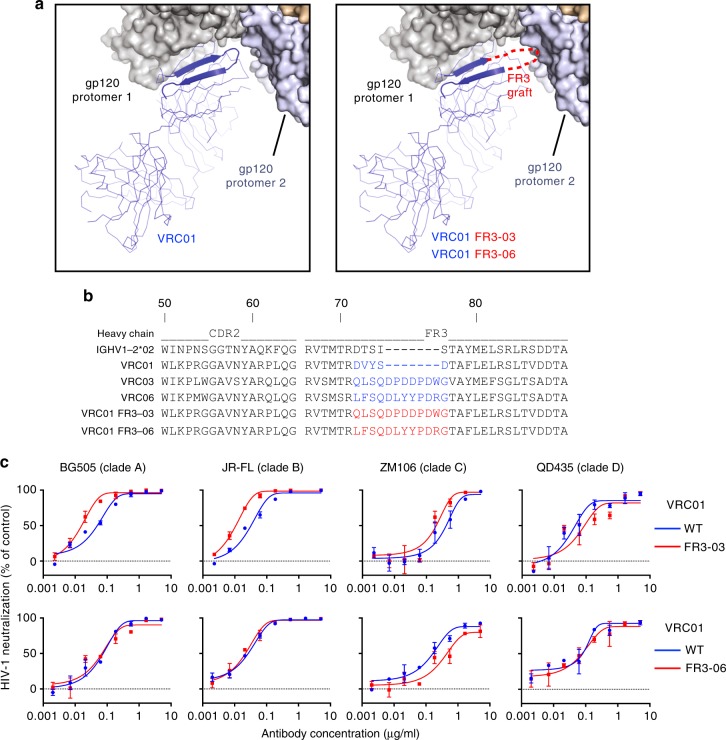
Engraftment of heterologous FR3 loops onto VRC01. **a** The elongated heavy chain FR3 loop of VRC03 and VRC06 (red sketch) was engrafted onto VRC01 to reach an adjacent gp120 protomer (protomer 2), creating two chimeric antibodies named VRC01 FR3-03 and VRC01 FR3-06, respectively. **b** Alignment of the amino acid sequence of the heavy chain CDR2 and FR3 regions from VRC01, VRC03, VRC06, and two chimeric antibodies where residues 73–77 of VRC01 (blue) were replaced with the extended FR3 loop of VRC03 or VRC06 (engrafted sequence highlighted in red). **c** HIV-1 neutralization by wild-type (WT) and chimeric antibodies tested on four HIV-1 strains of different clades (A–D) using the TZM-bl assay. Data represent the mean (±SD) from two replicate wells

Having demonstrated that contact with an adjacent gp120 protomer bolsters the trimer interaction of both CD4 and selected anti-CD4-BS antibodies, we hypothesized that conferring quaternary-contact capability might further enhance the function of already potent bNAbs that interact with a single gp120 protomer such as VRC01. Thus, we used existing high-resolution structures of gp120- or trimer-bound antibodies to rationally engraft the FR3 loops of VRC03 or VRC06 onto the shorter FR3 loop of VRC01 (aa 73–77), resulting in two chimeric antibodies that were designated VRC01 FR3-03 and VRC01 FR3-06, respectively (Fig. 2a, b). The chimeric antibodies were produced in HEK293FS cells and purified by affinity chromatography. All chimeric antibodies and their original forms showed a correct folding (Supplementary Fig. 3). Initial testing on a small panel of four HIV-1 strains from different clades demonstrated an increased neutralizing capacity of VRC01 FR3-03 against three of the four strains, while VRC01 FR3-06 did not show any improvement relative to the original antibody (Fig. 2c). These results indicated that the function of VRC01 was improved by engraftment of the FR3 loop from VRC03, but not from VRC06. We also tried engrafting of the CDR1 loop from VRC-CH31, which was shown to be functionally important^25^, onto the potent bNAbs N6 and VRC07-523-LS. However, both of the resulting chimeras showed a reduced neutralization capacity (Supplementary Fig. 1), suggesting a specific adaptation of the CDR1 loop to the VRC-CH31 antibody framework, which is incompatible with engraftment onto other antibodies.

**Fig. 3:**
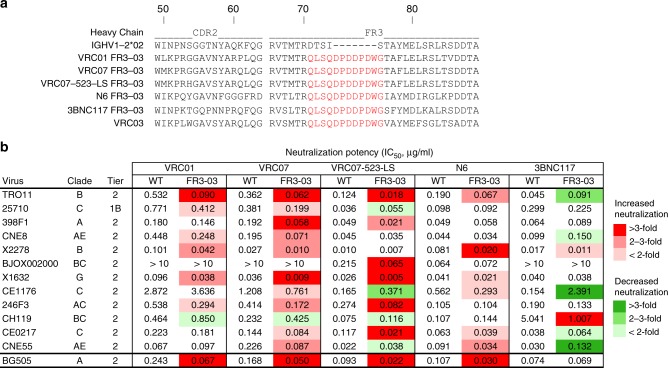
Engraftment of the VRC03 FR3 loop onto different HIV-1 bNAbs. **a** The FR3 loop of VRC03 (red) was engrafted onto five anti-CD4-BS bNAbs (i.e., VRC01, VRC07, VRC07-523-LS, N6, and 3BNC117), replacing their shorter FR3 loops. **b** A small global panel of HIV-1 strains^8^ was used to evaluate the neutralizing capacity of WT and chimeric antibodies using the TZM-bl assay. The IC_50_ values were interpolated from the dose–response curves against each virus and color-coded according to the fold change between the chimeric antibody and its respective WT form; different shades of red denote increased neutralization; different shades of green denote decreased neutralization. All neutralization assays were performed in duplicate wells

Next, we tested if the VRC03 FR3-loop engraftment could improve the neutralizing capacity of other anti-CD4-supersite antibodies with high potency and breadth. Thus, following the same strategy used for VRC01 FR3-03, we generated chimeric antibodies based on four additional bNAbs, namely, N6, VRC07, VRC07-523-LS, and 3BNC117 (Fig. 3a). The neutralizing potency of these chimeric antibodies and VRC01 FR3-03 was tested on a previously defined representative small global panel of 12 HIV-1 Envs^8^. All chimeric antibodies except 3BNC117 FR3-03 showed an increased neutralization potency against at least 50% of the 12 pseudoviruses, as well as against the BG505 Env used as a reference, with only occasional isolates showing a decreased potency; in contrast, chimerization of 3BNC117 resulted in decreased neutralization against 5 of the 12 Envs (Fig. 3b).

To investigate the mechanism underlying the increased neutralization potency of chimeric VRC01, VRC07, VRC07-523-LS, and N6, we measured the ability of the four chimeras and their unmutated counterparts to bind soluble Env trimers by surface plasmon resonance (SPR). MAb 2G12 was used to capture the BG505 SOSIP.664 soluble trimer, and the chimeric antibodies were used in the flow phase. SPR analysis showed increased binding for all the chimeric antibodies, compared to their original forms, except for 3BNC117, which was less potent than its original form (Supplementary Fig. 4a). The enhanced binding of chimeric antibodies was confirmed by ELISA (Supplementary Fig. 4b). As a control, we also tested binding of the chimeric antibodies to monomeric gp120 from isolates BG505 and BaL. Consistent with the lack of quaternary contact, all chimeric antibodies showed an unaltered or even slightly reduced binding capacity to gp120 monomers (Supplementary Fig. 4c, d).

As noted above, the FR3 loop of 3BNC117 is longer than that of N6, VRC01, and VRC07, and was shown by cryo-EM to contact the V3-loop base of an adjacent gp120 protomer in a complex with the BG505- SOSIP.664 trimer^9^. Thus, it is likely that engraftment of the VRC03 loop disrupted the pre-existing quaternary contact without replacing it with an equally effective paratope, suggesting that the 3BNC117 FR3 loop might be optimized for establishing quaternary contact. To validate this hypothesis, we introduced the FR3 loop of 3BNC117 (FR3-BNC) into VRC01, VRC07, VRC07-523-LS, and N6, but none of the chimeric antibodies showed an improved neutralization potency against the small global HIV-1 panel (Supplementary Fig. 5).

### Chimeric bNAbs have increased potency against global HIV-1 isolates

**Fig. 4:**
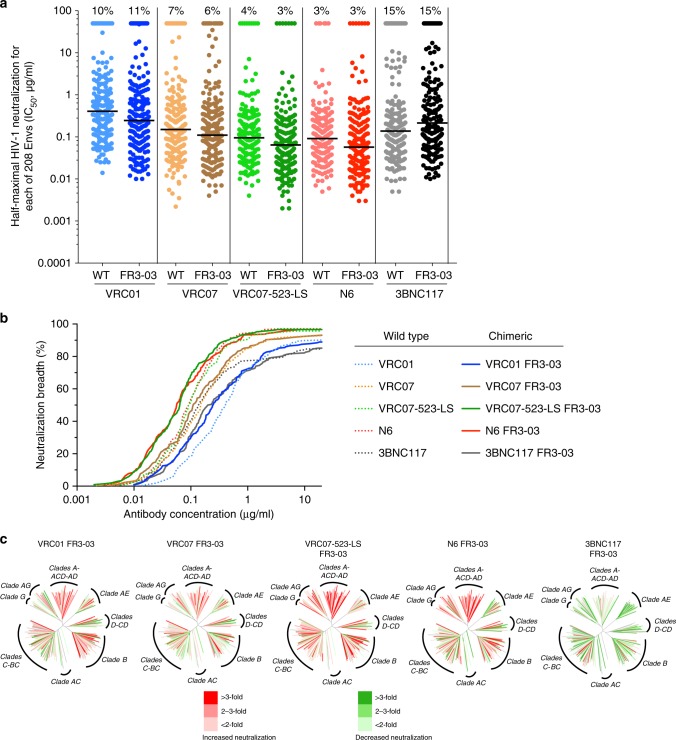
Increased potency of chimeric antibodies against global HIV-1 isolates. **a** Neutralization titers (IC_50_, μg/mL) for WT and VRC03 FR3-loop chimeric antibodies against a large global panel of 208 HIV-1 strains of different clades and circulating recombinant forms (CRF). The median IC_50_ value against antibody-sensitive strains is indicated by the horizontal line. All neutralization assays were performed in duplicate wells. The percentage of insensitive strains at doses up to 50 μg/mL is indicated on the top for each antibody. **b** Neutralization breadth for WT and chimeric antibodies as shown by plotting the number of sensitive isolates at increasing antibody concentrations. Chimeric antibodies are indicated by solid lines; WT antibodies by dotted lines. **c** Dendrograms show the fold change of each chimera’s neutralization potency against 208 HIV-strains, as compared to the original antibody

To verify the extent of neutralization enhancement by VRC03 FR3-loop-chimeric antibodies against global HIV-1 isolates, we tested their ability to neutralize a large panel of 208 Envs encompassing the most prevalent genetic subtypes of HIV-1 worldwide. In agreement with the results obtained on the small global panel, chimeric VRC01, VRC07, VRC07-523-LS, and N6 displayed an overall increased neutralization potency compared to that of the original antibodies, as shown by lower median IC_50_ and IC_80_ values, along with a similar or slightly increased breadth of activity (Fig. 4a, b). A large number of strains showed a >twofold increased susceptibility to chimeric bNAbs (Supplementary Fig. 6). The log_10_ of the median IC_50_ and IC_80_ values were significantly lower (*p* = 0.0026 and 0.0037, respectively, by two-tailed *t* test) for the chimeric antibodies vs. their original counterparts. Notably, the number of Envs that were neutralized with IC_50_ and IC_80_ values below 0.1 μg/mL by these four chimeric antibodies was significantly higher in comparison with neutralization by the original antibodies (*p* = 0.0079 and 0.0040, respectively, by two-tailed *t* test). The most effective chimeric antibodies were VRC07-523-LS FR3-03 and N6 FR3-03, which showed median IC_50_ values of 0.063 and 0.056 μg/mL, respectively (Supplementary Data 1 and 2). Detailed analysis of neutralization sensitivity among different genetic subtypes showed a higher increase in sensitivity to chimeric antibodies among clade-A, clade-B, clade-C, and clade AE strains, while clade-D and other mixed genotypes had lower degrees of improvement (Fig. 4c, Supplementary Fig. 7 and Supplementary Data 1 and 2). In contrast, as seen on the small global HIV-1 panel, chimeric 3BNC117 FR3-03 showed an overall decrease in neutralization potency on the large 208-Env panel compared to the original antibody (median IC_50_ and IC_80_, 0.149 and 0.466 μg/mL vs. 0.110 and 0.298 μg/mL, respectively).

### Sequence signatures of chimeric antibody sensitivity

Next, we performed a comprehensive comparative sequence analysis on the large 208-Env panel to identify signatures associated with sensitivity to neutralization by VRC03 FR3-loop-chimeric antibodies. Analysis of Envs showing >1.5-fold change in sensitivity to FR3-loop chimeras vs. wild-type (WT) antibodies identified two major hot spots significantly associated with sensitivity to more than one chimeric antibody: a region located distal to CD4-BS2, centered around the base of the V3 loop, and a region centered on the stem of the V4 loop (Supplementary Fig. 8a). While the V4-stem region is distant from the antibody-contact surface and its potential role remains obscure, the area around the V3 base, which also includes residues in contiguous areas of C4 and V2, appears to be directly involved in contact with the engrafted FR3 loop. The most prominent site, which appeared among the top 20 associations for all four chimeric antibodies with improved neutralization, was aa 318 at the base of V3, where a tyrosine was highly favorable for increased sensitivity while phenylalanine was unfavorable (Supplementary Fig. 8a and Supplementary Data 3). In a cryo-EM structure of VRC03 bound to a soluble Env trimer, Y318 appears to be involved in the quaternary contact with the extended FR3 loop, along with R304, also at the base of V3, which appeared among the top 20 associations for VRC01 and VRC07-523-LS and among the top 100 for all chimeric antibodies except 3BNC117. Additional associations included aa 429 and 440 in the C4 region, which may influence the orientation of FR3-contacting loops, as well as residues in the V2V3-loop region that appear to stabilize the β-sheet arrangement of the loops at the trimer apex (e.g., aa 161, 165, 174, and 321). Although none of the residues involved in CD4-BS2 (i.e., E62, E64, H66, and K207) emerged among the top 100 associations, it has to be emphasized that three of them are absolutely conserved among the 208 HIV-1 isolates and therefore no sequence associations could be revealed by this analysis; however, two contiguous residues, aa 63 and 65, were significantly associated with sensitivity to more than one chimeric antibody. A strong association restricted to N6 was aa 276, an N-glycosylation site, where an asparagine was highly associated with improved neutralization, while an aspartic acid that impaired glycosylation had the opposite effect. Interestingly, there was no overlap between the top-ranked associations for 3BNC117 FR3-03 and the other four chimeric antibodies (Supplementary Data 3), further reinforcing the unique quaternary interaction of this antibody with the HIV-1 Env trimer.

### Co-crystal structures of chimeric antibodies with a soluble trimer

**Fig. 5:**
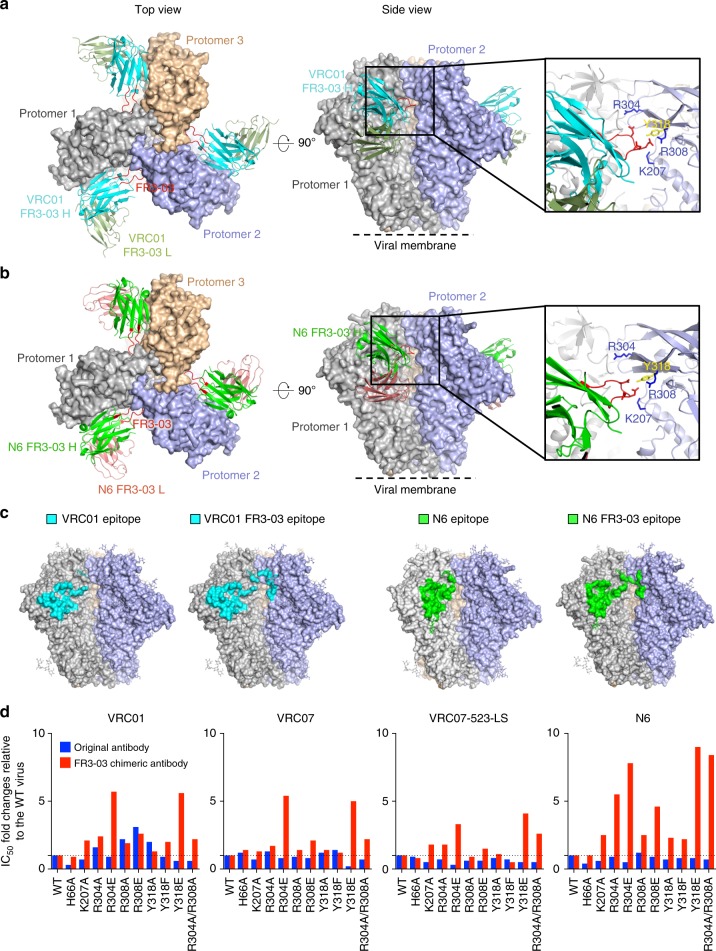
Crystal structure and epitope mapping of two FR3-loop-chimeric antibodies. **a**, **b** Crystal structures of chimeric antibodies VRC01 FR3-03 (PDB ID: 6NNF) (**a**) and N6 FR3-03 (PDB ID: 6NM6) (**b**) single-chain variable domain (scFv) complexed with a soluble, truncated and stabilized Env trimer (BG505 SOSIP.664). The elongated heavy chain FR3 loop appears stretched outwards to reach the V3 base and part of the CD4-BS2 of a neighboring protomer (protomer 2). The quaternary contact with the engrafted FR3 loop (red) appears to involve direct interaction with residues K207, R304, R308, and Y318 in protomer 2, as shown in the close-up view. **c** The WT and chimeric VRC01 and N6 footprints on the Env trimer are highlighted in turquoise and greeen, respectively. **d** Mapping of the binding interface of the engrafted FR3 loop by mutagenesis. Neutralization sensitivity of pseudoviruses bearing mutated BG505 Env to WT and chimeric antibodies, as assessed using the TZM-bl assay. All neutralization assays were performed in duplicate wells. The fold change in IC_50_ between each mutant and the WT virus (set to 1) was calculated

To gain structural insights into the interaction of the chimeric antibody with the HIV Env trimer, we solved the crystal structures of chimeric antibodies VRC01 FR3-03 (PDB ID: 6NNF) and N6 FR3-03 (PDB ID: 6NM6) complexed with the BG505 SOSIP.664 trimer (Fig. 5a, b and Supplementary Table 1). In both structures the elongated FR3 loop is well defined and shows a conformation similar to that of the FR3 loop in a trimer-bound VRC03 cryo-EM structure^18^ (Supplementary Fig. 9). The overall orientation of VRC01 FR-03 and N6 FR3-03 is very close to that of the respective original antibodies (Fig. 5a, b, Supplementary Fig. 9). While the binding footprints of both chimeric antibodies on the first gp120 protomer are nearly identical to those of the original antibodies, the FR3 loop-contact surface is mainly located on the neighboring protomer (Fig. 5c), indicating that the elongated FR3 loop establishes quaternary contacts without affecting the “classic” interaction. Besides contacting CD4-BS2, mostly through residue K207, the FR3 loop seems to bind residues with long side chains at the base of the V3 loop: the aromatic Y318 and the positively charged R304 and R308 (Fig. 5a, b). Of note, two of these three residues were significantly associated with sensitivity to chimeric antibodies in the analysis presented in Supplementary Data 3. These observations indicate that, by establishing quaternary contact, the engrafted FR3 loop expands the antibody-contact surface on the trimer, thereby stabilizing the interaction. The fact that the FR3 loop in the two structures adopts essentially the same conformation suggests that the specific conformation of the VRC03 FR3 loop can be accommodated onto different antibody frameworks of the VRC01 class to achieve quaternary interaction with the trimer.

### Mapping of the FR3-loop-interactive surface by mutagenesis

To verify the importance of the quaternary interaction between the engrafted VRC03 FR3 loop and the neighboring gp120 protomer, we mutated key residues identified as potentially interactive with this loop based on the crystal structure and previous mutagenesis, namely, H66 and K207 in CD4-BS2, and R304, R308 and Y318 in the V3-loop base. Pseudoviruses bearing the WT and mutated Envs were produced and tested for their sensitivity to neutralization by all chimeric vs. original bNAbs except 3BNC117. Alanine mutations in CD4-BS2 (H66A, K207A) induced a moderate reduction of sensitivity to the chimeric antibodies, particularly K207A with VRC01 FR3-03 and N6 FR3-03 (Fig. 5d and Supplementary Table 2), while charge inversions at the base of the V3 loop induced more pronounced changes. Specifically, the introduction of negative charges at positions 304 and 318 strongly reduced the sensitivity to all chimeric antibodies, indicating that these chimeras share a common FR3-03 binding mode. Other mutations in the V3 had diverse impact on different chimeras (Fig. 5d and Supplementary Table 2). Of note, N6 FR3-03 was sensitive to mutations in most of the tested sites. Mutations to alanine had generally less pronounced effects than charge inversions, suggesting a dominant role of electrostatic interactions; however, simultaneous alanine mutation of R304 and R308 yielded a pronounced loss of neutralization, particularly for chimeric N6 (Fig. 5d and Supplementary Table 2). Unfortunately, charge inversions in CD4-BS2 could not be tested because their infectivity is completely abrogated^13^. Although the role of subtle conformational alterations upon mutagenesis can not be ruled out, these data confirmed the involvement of residues in CD4-BS2 and at the base of the V3 loop in FR3-loop contact, as observed in the two crystal structures. Thus, long-range charge interactions between R304/R308/Y318 and the negatively charged FR3 loop appear to play a critical role in the enhanced trimer interaction of chimeric antibodies.

### FR3-loop chimerization reduces autoreactivity

Several anti-HIV-1 antibodies possess different degrees of autoreactivity, which can limit their use in HIV-1 prevention or treatment^10^, and induction of autoreactivity is a risk associated with ex vivo antibody engineering. Thus, we evaluated whether chimerization by FR3-loop engraftment had any effects on autoreactivity. Reactivity with two autoantigenic targets were tested: antinuclear antibodies (ANA), as detected by Hep2 cell staining, and anticardiolipin antibodies, as detected by ELISA. Strikingly, all the chimeric antibodies showed a reduced autoreactivity in the ANA system, compared to their original form (Table 1 and Supplementary Fig. 10). In particular, N6 and VRC07, which in their unaltered form were mildly reactive in the ANA test, lost all detectable reactivity after chimerization, while VRC07-523-LS, which was clearly ANA-reactive in the unaltered form, showed only mild reactivity after chimerization. None of the anti-CD4-BS bNAbs, whether WT or chimeric, showed autoreactivity in the anticardiolipin assay. These results indicate that engraftment of the VRC03 FR3 loop altered an autoreactive domain in these antibodies, making the chimeric antibodies potentially safer than their original counterpart for in vivo use.

**Table 1:** Autoreactivity (ANA and anticardiolipin) of WT and chimeric antibodies

Antibody	Wild-type (ANA)	FR3-03 chimera (ANA)	Wild-type (anticardiolipin)	FR3-03 chimera (anticardiolipin)
VRC01	Not reactive	Not reactive	Not reactive	Not reactive
VRC07	Mildly reactive	Not reactive	Not reactive	Not reactive
VRC07-523-LS	Autoreactive	Mildly reactive	Not reactive	Not reactive
N6	Mildly reactive	Not reactive	Not reactive	Not reactive
3BNC117	Mildly reactive	Not reactive	Not reactive	Not reactive
VRC03	Not reactive	n.a.	Not reactive	n.a.
VRC01-LS	Not reactive	n.a.	Not reactive	n.a.
VRC07-G54W	Autoreactive	n.a.	Mildly reactive	n.a.
4E10	Autoreactive	n.a.	Autoreactive	n.a.

### FR3-loop chimerization prolongs antibody in vivo half-life

**Fig. 6:**
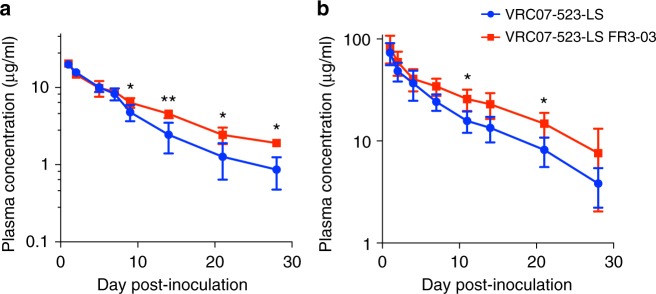
Prolonged in vivo half-life of an FR3-loop chimeric antibody. **a** The pharmacokinetics of WT and chimeric VRC07-523-LS were evaluated in human FcRn-transgenic mice (*n* = 5 in each group). **b** The pharmacokinetics of chimeric VRC07-523-LS were evaluated in naïve rhesus macaques (*n* = 3), and compared to data obtained for the WT antibody in a previous study (*n* = 4)^20^. Antibodies at 5 mg/kg body weight for mice and 10 mg/kg for monkeys were administered intravenously, and plasma antibody levels were measured by ELISA using a gp120 resurfaced core antigen (RSC3)^20^. The mean ± SD concentration at each time point is plotted. *P < 0.05; **P < 0.01 by unpaired two-sided *t* test

To evaluate if chimerization by engraftment of the VRC03 FR3 loop had an effect on antibody pharmacokinetics in vivo, we studied the half-life of the chimeric VRC07-523-LS, compared to its original counterpart, in human FcRn-transgenic mice. The antibody concentration in plasma was measured at different time points over a period of 28 days after inoculation. The persistence of the chimeric antibody in plasma of injected mice was extended, with significantly higher plasmatic concentrations at all time points after day 7 postinoculation; the mean plasma concentration on day 14 postinoculation was 4.57 ± 0.55 µg/mL (±values represent the standard deviation) for VRC07-523-LS FR3-03, as compared to 2.45 ± 1.05 µg/mL for the unmodified antibody (Fig. 6a).

Next, we evaluated the in vivo pharmacokinetics of chimeric VRC07-523-LS in three naïve rhesus macaques, comparing the results to previously reported data for the unmodified antibody^20^. The mean plasma concentrations for the two antibodies started to diverge on day 7 postinoculation, and on day 21 they were 14.80 ± 4.03 µg/mL for VRC07-523-LS FR3-03 vs. 8.18 ± 2.63 µg/mL for the unmodified antibody (*p* < 0.05; Fig. 6b). Since the plasma half-life of antibodies was shown to be shortened by autoreactivity^12^, the prolonged half-life measured for our chimeric antibody was likely due, at least in part, to its reduced autoreactivity.

### FR3-loop insertion frequency in infected and uninfected individuals

In-depth analysis of the immunoglobulin VH1–2 gene family by next-generation sequencing (NGS) using VH1-specific primers (Supplementary Table 3) revealed a high frequency of heavy-chain FR3-loop insertions (4.31% of total VH1–2 reads) in peripheral blood of the original patient from whom VRC01 and VRC03 were derived (donor 45)^16^, with peaks at 4- and 7-residue insertions (1.03% and 1.39%, respectively), while deletions were rare (0.25%) in this region (Table 2). To investigate the prevalence of heavy-chain FR3-loop insertions in individuals without HIV-1 infection, we analyzed peripheral blood from six healthy, HIV-seronegative donors. The overall numbers of VH1–2 reads were markedly lower in these donors, presumably due to a lack of expansion of VH1–2-expressing B cells and in part to the fact that NGS was performed using nonspecific rather than VH1-specific primers. The frequency of FR3-loop insertions was relatively high in three healthy donors (7.93%, 1.58%, and 1.46% of total VH1–2 reads, respectively), but the vast majority were one-residue insertions except in one donor who had a significant number of three-residue insertions; the other three donors had low overall frequencies of FR3 insertions (<0.5%), but in two of them (donors 4 and 5) there was a greater proportion of two- or more aa insertions (Table 2). These data confirm the intensiveness of the antibody affinity maturation process in an HIV-infected individual (donor 45), which results not only in somatic hypermutations, but also in large insertions (>3 aa) that may expand the epitope specificities of anti-Env antibodies. Despite a lower frequency, FR3 insertions also occur in healthy seronegative donors, suggesting that FR3 insertions may be used by the adaptive immune system to expand the epitope reach of a diverse range of antibodies.

**Table 2:** Frequency of FR3 insertions/deletions in an HIV-seropositive patient (donor 45, the original source of VRC01-class antibodies) and six healthy donors

Number of inserted aa. on FR3	HIV+ donor 45	Healthy donor 1	Healthy donor 2	Healthy donor 3	Healthy donor 4	Healthy donor 5	Healthy donor 6
0	1,109,750	1966	14,483	5529	8362	37,861	2418
1	962	167	170	81	8	10	7
2	1557	3	1	1	3	12	3
3	8472	0	61	0	0	1	1
4	11,959	0	0	0	15	5	0
5	8330	0	0	0	8	4	1
6	71	0	1	0	0	3	0
7	16,142	0	0	0	0	0	0
8	2584	1	0	0	0	0	0
9	4	0	0	0	0	0	0
10	1	0	0	0	0	1	0
>10	4	0	0	0	0	3	0
Deletion	2903	20	0	0	23	33	3
Total VH1–2 Reads	1,162,739	2157	14,716	5611	8419	37,932	2433
Freq. of insertion	4.31	7.93	1.58	1.46	0.4	0.1	0.49
Freq. of deletion	0.25	0.93	0	0	0.27	0.09	0.12

## Discussion

The present study illustrates a strategy to improve the biological properties of natural antibodies by engraftment of new functional elements derived from other antibodies. We previously found that selected patient-derived antibodies, such as VRC03 and VRC06, are able to reach the second, quaternary CD4-BS through unusually long heavy-chain FR3 loops^13^. Herein, we provide formal proof for the functional role of these extended FR3 loops, showing that their deletion dramatically reduces the ability of VRC03 and VRC06 to bind to the Env trimer and neutralize HIV-1 infectivity. In a previous study, VRC03 and VRC06 were shown to have an uncommon dual specificity not only for the CD4-BS but also for the coreceptor-binding site in gp120, although no attempts were made to define the role of quaternary contacts^7^. Unlike VRC03 and VRC06, the more potent VRC01-class bNAbs such as VRC01, VRC07 and N6 appear to interact primarily, if not exclusively, with a single gp120 protomer and possess shorter FR3 loops that marginally contribute to antigen binding. Based on these premises, in the present study we rationally designed an FR3-loop grafting strategy in an attempt to confer on several potent CD4-BS bNAbs the ability to interact with additional target epitopes on a neighboring protomer of gp120.

While engraftment of the FR3 loop of VRC06 or the CDR1 loop of VRC-CH31, another antibody with an uncommon loop extension in its heavy chain variable region^25^, yielded no significant functional benefit, the introduction of the VRC03 FR3 loop significantly improved the functionality of four potent VRC01-class bNAbs, namely, VRC01, VRC07, VRC07-523-LS, and N6, without affecting their recombinant production efficiency. In contrast, another potent bNAb, 3BNC117, was not improved. The reasons for this discrepancy remain unclear, although the native FR3 loop of 3BNC117 is longer than that of VRC01, VRC07, and N6, and was previously shown by cryo-EM to establish quaternary interaction, reaching the V3-loop base of an adjacent gp120 protomer^9^. The FR3 loop of the nearly identical bNAb 3BNC60 was reported to be essential for maintaining the neutralizing capacity of this bNAb, and its insertion into a weakly neutralizing antibody derived from the same patient, 3BNC55, increased its neutralizing activity^24^. Thus, although VRC03 and 3BNC117 belong to the same antibody family, it is likely that the engrafted VRC03 FR3 loop disrupted the pre-existing quaternary contact of 3BNC117 but did not adopt the proper orientation for establishing and effective interaction with the adjacent gp120 protomer.

We obtained the crystal structures of two chimeric antibodies, VRC01 FR3-03 and N6 FR3-03, in complex with the BG505 SOSIP.664 soluble trimer, which confirmed the quaternary contacts established between the engrafted FR3 loops and a second gp120 protomer. Interestingly, we found that the FR3 loop-contacting region comprises not only part of CD4-BS2, but also elements at the base of the V3 loop, which form a contiguous surface distal to CD4-BS2 in the neighboring protomer. Both structural analysis and mutagenesis studies suggest that binding of the FR3 loop is mainly mediated by long-range electrostatic interactions between negatively charged aspartate residues in the FR3 loop and positively charged residues in gp120. From the crystal structure, Y318 seems to establish the main direct contact with the tip of the FR3 loop, but neighboring basic residues in V3, such as R304 and R308, are also favorably positioned and point toward the incoming FR3 loop. The role of these residues was confirmed by mutagenesis studies in which the introduction of negative charges resulted in loss of neutralization by chimeric antibodies, as well as by sequence analysis of sensitive strains in the large 208-Env global panel. Thus, it appears that the quaternary contact provided by the FR3 loop stabilizes the antibody binding to the Env trimer, leading to increased neutralization potency.

Another significant, as much as unexpected, benefit of VRC03 FR3-loop engraftment was a dramatic reduction in autoreactivity of all the engineered bNAbs. Autoreactivity is an inherent property of certain anti-HIV-1 antibodies, likely related to the close association of viral and self epitopes, especially in proximity of the viral membrane, which can pose severe limitations to the use of antibodies in therapy and prevention^10^. Induction of autoreactivity is also an important risk linked to modification of natural antibodies. For example, mutation of G54 in the CDR2 domain of VRC01-family bNAbs to residues with bulky hydrophobic rings was reported to enhance autoreactivity^20^. Indeed, VRC07-523-LS, a more potent modified version of VRC07 bearing a histidine at position 54, is the most autoreactive among the VRC01-class antibodies currently in the pipeline for potential clinical use (Phase 1 clinical trial: VRC605). Likewise, our results showed that N6, which naturally has a tyrosine at position 54, is mildly autoreactive. The reason why engraftment of the VRC03 FR3 loop strongly reduced autoreactivity in all the antibodies tested is still unclear. Given the spatial proximity between the FR3 loop and CDR2, it is possible that implantation of an extended FR3 loop may conceal the self-reactive paratopes. Corroborating this hypothesis, VRC03 is not autoreactive despite possessing a tryptophan at position 54. Linked to the reduction of autoreactivity, we found that the in vivo persistence of chimeric VRC07-523-LS FR-03 in humanized FcRN mice and rhesus macaques was prolonged, with significantly higher plasmatic concentrations compared to the unmodified antibody after the first week postinoculation. The prolonged half-life documented both in transgenic mice and in nonhuman primates represents an important benefit for the potential in vivo utilization of bNAbs in human therapy and prevention.

Chimerization of anti-HIV-1 antibodies has been used to create bispecific or trispecific molecules with improved neutralization potency and breadth^26–29^, which have shown enhanced in vivo protection against HIV-1 or SHIV challenges in animal models^28,29^. Our strategy differs from these previous attempts because we created chimeric antibodies by engrafting a single heterologous domain from another natural antibody with minimal modifications of the heavy chain, rather than recombining the whole antigen-binding fragment. This approach does not alter the overall configuration of the parental antibody, as confirmed by our crystal structures, which likely contributed to maintaining the production of our chimeric antibodies as efficient as that of their original forms without yielding undesired side products. For the same reasons, our chimeras maintained their original target specificity. Moreover, the engraftment of a natural sequence from another human antibody in its original spatial position minimizes the risk of inducing antidrug antibodies. Owing to their increased potency and reduced autoreactivity, FR3-loop chimeric antibodies may thus be valuable candidates for use in HIV-1 prevention and treatment.

Although the present study was focused on a single antibody family, the strategy of structure-based paratope engraftment described herein might be applicable to other antibody families both within and outside the HIV field. The concept of targeting the heavy-chain FR3 loop for antibody engineering was first proposed in 1992^30^. Notably, a potent anti-HIV-1 bNAb of a different class and directed against a different supersite of vulnerability, 35O22, also possesses a heavy-chain FR3-loop insertion that participates in antigen recognition^2^, and a functionally relevant FR3 insertion was described in a neutralizing antibody against a different infectious agent, influenza virus^17,31^. Moreover, our NGS analysis of healthy donors indicated that heavy-chain FR3-loop insertions do occur in HIV-seronegative individuals, suggesting that this strategy is used by the immune system to expand the epitope specificity of different antibody families. It is remarkable that while members of the same multidonor antibody class share the same mode of antigen recognition and similar B-cell ontogeny, different developmental pathways can achieve recognition of conserved surfaces that neighbor the core site of recognition on the HIV-1 Env trimer. Some of these differences are apparent when comparing VRC01-class antibodies derived from different donors, such as the difference between the heavy-chain CDR1 region of VRC01 and VRC-CH31, with the latter possessing a unique insertion that allows recognition of CD4-BS2. However, changes are also observed within members of the same lineage; for example, analysis of donor 45^16^, the source of VRC01, shows how a highly differentiated lineage can develop different types of extended loops, not limited to the FR3 insertion that allows VRC03 and VRC06 to recognize CD4-BS2. For example, different evolutionary pathways yielded extended heavy-chain CDR3 loop in VRC08 (14 amino acids longer than in VRC01), which potentially makes contacts with the α0 helix in the neighboring gp120 protomer^16^. These different solutions leading to an extended epitope recognition highlight the stochastic nature of indels in antibody maturation, with the FR3 chimeras described here demonstrating how new paratope engraftment can not only improve neutralization breadth and potency, but also impact antibody polyreactivity and in vivo half-life. In light of these considerations, it is conceivable that not only FR3-loop but also other indel engraftments could be used to add new functionalities to antibodies directed against diverse antigenic targets.

## Methods

### Mutagenesis

Site-directed mutagenesis was carried out with Q5^®^ Site-Directed Mutagenesis Kit (New England BioLabs Inc.; E0554S) for antibody-encoding plasmids and the QuikChange II site-directed mutagenesis kit (Agilent Technologies; 200524) for Env-encoding genes. A full list of DNA primers used for mutagenesis is provided in Supplementary Table 4.

### HIV-1 Env trimer expression and purification

The soluble BG505 and JRFL SOSIP.664 were expressed in HEK293FS (human embryonic kidney cells, female, obtained from ThermoFisher) and purified with a *Galanthus nivalis* lectin column (Vector laboratories). The eluted proteins were first dialyzed to PBS, followed by two rounds of size-exclusion chromatography. A further cleanup step with 447-52D affinity column was performed. Proteins were concentrated and stored in PBS supplemented with 10% glycerol at −80 °C.

### Antibody expression and purification

All antibodies were expressed in HEK293FS cells by co-transfecting equal amount of heavy chain and light chain plasmids. FreeStyle^™^ MAX Reagent (Thermo Fisher Scientific; 16447500) was used for transfection according to manufacturer’s protocol. Cell-free supernatants were collected after 5-day incubation at 37 °C and filtered through a 0.22-μm filter. Antibody was purified by passing the supernatants through protein A column. After washing with PBS, protein was eluted with low-pH IgG elution buffer (Thermo Fisher Scientific; 21009), followed by neutralization with 100 mM Tris-HCl pH 9.0. The eluted protein was dialyzed to PBS and stored at 1 mg/ml at −80 °C.

### Surface plasmon resonance analyses

Surface plasmon resonance analyses were performed at 25 °C on a Biacore 3000 (GE Healthcare) instrument with HBS-EP+ Buffer (GE Healthcare, BR100826). Mab 2G12 was diluted to 20 μg/ml in 10 mM sodium acetate pH 5.0 and immobilized onto two flow cells of a CM5 sensor chip to ~7000 response units (RU) using Amine Coupling Kit (GE Healthcare; BR100050). Purified BG505 SOSIP.664 at 400 nM was injected to the sample flow cell at a flow rate of 6 μl/min for 100 s. The capture amount was 710–770 RU. The other flow cell was used as reference. WT or chimeric bNAbs were injected to both the reference and sample flow cells at 400 nM at a flow rate of 30 μl/min for 3 min, followed by a 6-min dissociation phase. HBS-EP+ injections were also performed as blank sensorgrams. The binding curve was adjusted by subtracting the blank sensorgram in BIAevaluation software and plotted in Prism 7.

### Autoreactivity analysis

Autoreactivity was determined by ANA Hep-2 Staining Analysis (ZEUS Scientific Cat. No: FA2400) and anticardiolipin ELISA (Inova Diagnostics Cat. No.: 708625). All antibodies were tested at 25 and 50 μg/ml as per manufacturer’s protocols. Scores from 0 to 3 were defined with four control antibodies VRC01-LS, 4E10, VRC07-523-LS, and VRC07-G54W. Hep-2 cells (human malignant epithelial cells, female, obtained from ZEUS Scientific). Signals of the test antibody were visually estimated in comparison to the control ones. Scores greater than 1 at 25 μg/ml were classified as autoreactive, and between 0 and 1 as mildly autoreactive. In the cardiolipin ELISA, bNAbs were tested at a starting concentration of 100 μg/ml, followed by 3-fold dilutions. IgG phospholipid (GPL) units were calculated from the standard curve. GPL score < 20 was considered as not reactive, 20–80 as low positive and >80 as high positive.

### In vivo pharmacokinetics

Human FcRn-transgenic mice (*n* = 5 per antibody) were used to assess the pharmacokinetics of the WT and chimeric VRC07-523-LS antibody. Each animal was infused intravenously with 5 mg mAb/kg of body weight. Whole blood samples were collected at day 1, 2, 5, 7, 9, 14, 21, and 28. Plasma was separated by centrifugation. Plasma mAb levels were measured by ELISA using a gp120 resurfaced core antigen (RSC3)^20^. All mice were bred and maintained under pathogen-free conditions at an American Association for the Accreditation of Laboratory Animal Care (AAALAC)-accredited animal facility at the NIAID and housed in accordance with the procedures outlined in the Guide for the Care and Use of Laboratory Animals. All mice were between 6 and 13 weeks of age. The study protocol was evaluated and approved by the NIH Animal Care and Use Committee (ASP VRC-18-747).

Naïve rhesus macaques (*n* = 3) were infused with 10 mg/kg of the chimeric VRC07-523-LS antibody. Whole-blood samples were collected prior to injection, and at days 1, 2, 5, 7, 11, 14, 21, and 28 after injection. Serum was separated by centrifugation after clotting of the blood and kept frozen until use. Serum samples were heat inactivated at 56 °C for 60 min, and lipoproteins were pelleted. Plasma mAb levels were measured by ELISA using RSC3^20^. All animal experiments were reviewed and approved by the Animal Care and Use Committee of the Vaccine Research Center, NIAID, NIH, and all animals were housed in a fully AAALAC-accredited facility with stringent standard operating procedures and compliant with U.S. Animal Welfare Act (AWA) and Regulations, the Public Health Service (PHS) Policy on Humane Care and Use of Laboratory Animals, the Guide for the Care and Use of Laboratory Animals and all applicable NIH Policies on in vivo research.

### Enzyme immunoassays

To test the binding of antibodies to SOSIP.664 trimers or gp120 monomers, 96-well ELISA plates (Corning) were coated with 5 μg/ml of *G. nivalis* lectin (Sigma; L8275-5MG) at 4 °C overnight. Plates were washed three times with 1× wash buffer (R&D Systems) and blocked with 0.2% casein in PBS. The same washing procedure was carried out after each incubation. The SOSIP.664 trimer or gp120 monomers was added at 1 or 2 μg/ml and incubated at RT for 1 h. Serial dilutions of WT or chimeric antibodies were added to duplicate wells for 1 h at RT, followed by incubation with horseradish peroxidase-conjugated goat anti-human IgG (Sigma; A8419 or Jackson ImmunoResearch; 109-035-008). Substrate reagent and stop solution (R&D Systems) were used to reveal the signal. Light absorption at 450 nm was measured with a luminometer (PerkinElmer). All samples were tested in duplicate.

### Neutralization assays

HIV-1 pseudoviruses were produced in HEK293T cells by cotransfection of a backbone plasmid, pSG3^ΔENV^ and Env-expressiong plasmids in sixwell plates. TransIT^®^-293 Transfection Reagent (Mirus) was used according to the manufacturer’s protocol. The neutralizing activity of each antibody was measured by a single-round infection assay in TZM-bl cells (human malignant epithelial cells, female, obtained from the NIH AIDS Reagent Program). Pseudoviruses were incubated with serial dilutions of the antibody in duplicate wells of a 96-well plate for 30 min at room temperature, followed by addition of 10,000 TZM-bl cells/well. After 48 h incubation, a luciferase assay kit (Promega) was used to detect the luminescence signal. The antibody concentrations that yielded 50 or 80% neutralization were calculated by Prism 7. For the large panel of 208-Env pseudoviruses, neutralization assays were performed as described previously^32^.

### Sequence analysis of neutralization-associated signatures

We calculated the associations between sequence variability of full HIV-1 Env sequences and neutralization data from the 208-pseudovirus panel^33^ using an in-house version of the approach implemented in the R package SeqFeatR^34^. The neutralization data were preprocessed by retaining only the subset of Envs that showed either a decrease or an increase in neutralization potency by 1.5-fold or greater between the chimeric antibodies and their respective WT version. The resulting *p* values were corrected using the Holm multiple-testing method.

### Crystal structure determination

Purified BG505 SOSIP.664 trimers were mixed with the chimeric anti-CD4-BS antibody, a variant of 3H + 109L Fab and a variant of 35O22 scFv at a (1: 3.2: 3.2: 3.2) molar ratio (gp140 trimer: chimeric antibody: Fab: scFv) and incubated overnight at RT. The 3H + 109L variant contained two methionine substitutions in the light chain and the 35O22 variant contained three threonine and two serine mutations in the heavy chain. These alterations were designed to enhance the crystallization lattice^35^. Complexes were further purified by SEC and concentrated to 10–20 mg/ml for crystallization. Crystals of the N6 FR3-03 chimeric antibody (scFv) in complex with BG505 SOSIP.664, 3H + 109L Fab and 35O22 scFv were grown in 60 mM acetate pH 4.5, 420 mM sodium formate, 5% PEG 3,350 and 60 mM CaCl_2_ with a protein to reservoir ratio of 0.5–0.5 μl. Crystals were harvested and incubated in crystallization condition supplemented with 15% 2R,3R butanediol as cryo-protectant for 30 s before freezing in liquid nitrogen. Diffraction data was collected at SER-CAT beamline of the Advanced Photo Source at the Argonne national lab. HKL-2000 was used to process the diffraction data, followed by anisotropy data truncation by the UCLA anisotropy server (https://services.mbi.ucla.edu/anisoscale/). A structure of BG505.SOSIP.664 in complex with PGT122 and 35O22 (PDB ID: 4TVP) was modified by using Sculptor to change PGT122 Fab to 3H + 109L Fab. Molecular replacement was carried out using the modified 4TVP.pdb and the WT N6 as search models in Phaser. The transplanted framework loop from VRC03 can be clearly visualized in the resulting electron density map. Refinement and structure evaluation were carried out in Phenix.

Crystals of the VRC01 FR3-03 chimeric antibody (scFv) in complex with BG505 SOSIP.664, 3H + 109L Fab and 35O22 scFv were grown in 72 mM imidazole pH 6.5, 72 mM MgCl_2_, 0.96 M NaCl and 9.6% PEG 3350 with a protein to reservoir ratio of 0.5–0.5 μl. Crystals were harvested and incubated in crystallization condition supplemented with 15% 2R,3R butanediol as cryo-protectant for 30 s before freezing in liquid nitrogen. Crystals of the WT CH31 scFv in complex with BG505 SOSIP.664, 3H + 109L Fab and 35O22 scFv were grown in 90 mM imidazole pH 6.5, 90 mM MgCl_2_, 0.6 M NaCl, and 6% PEG 3350 with a protein to reservoir ratio of 0.5–0.5 μl. Crystals were harvested and incubated in crystallization condition supplemented with 15% 2R,3R butanediol as cryo-protectant for 30 s before freezing in liquid nitrogen. VRC01 FR3-03 complex structure and WT CH31 scFv complex structure were determined and refined using the same approach as for the N6 FR3-03 complex structure described above.

### Frequency of insertions into the heavy-chain FR3 region

The 454-pyrosequencing datasets from HIV-1 seropositive donor 45^16^ and healthy seronegative donors^36–38^ were downloaded from Short Read Archive (Supplementary Table 2). To evaluate the frequency of amino acid insertions into the FR3 region, the analysis was restricted to IgG genes. Standalone IgBlast (version 1.8) was used to assign V(D)J germline genes with IMGT scheme^39^, and the quality filter was used to keep the sequences with no stop codon, productive, and longer than 300 base pairs for the analysis. Reads containing the heavy-chain germline gene VH1–2 were selected to extract FR3 sequences. The length of the FR3 region from the different samples was compared to the length of the FR3 region from the VH1–2 germline gene. Since NGS samples from various studies used different primers for 454-pyrosequencing, we used the number of VH1–2 reads as the denominator to calculate the frequency of amino acid insertions into FR3 region.

## Supplementary information

Supplementary Information

Peer Review File

Description of Additional Supplementary Files

Supplementary Data 1

Supplementary Data 2

Supplementary Data 3

Reporting Summary

## Data Availability

All data generated in this study are available within this paper and the supplementary files. Crystal structure coordinates have been deposited into the PDB Protein Data Bank under accession codes: 6NNF, 6NM6 and 6NNJ.
