# Supplementary material for: Improvement of antibody functionality by structure-guided paratope engraftment
Source: Nat Commun. 2019 Feb 13;10:721. doi: 10.1038/s41467-019-08658-4 (PMC6374468; doi:10.1038/s41467-019-08658-4)
Supplement: Supplementary file 1 — Supplementary Information [file 41467_2019_8658_MOESM1_ESM.pdf]

nature COMMUNICATIONS

Supplementary Information for:

Improvement of Antibody Functionality by Structure-Guided Paratope Engraftment

by Qingbo Liu, Yen-Ting Lai, Peng Zhang, Mark K. Louder, Amarendra Pegu, Reda Rawi, Mangaiarkarasi Asokan, Xuejun Chen, Chen-Hsiang Shen, Gwo-Yu Chuang, Eun Sung Yang, Huiyi Miao, Yuge Wang, Anthony S. Fauci, Peter D. Kwong, John R. Mascola and Paolo Lusso

<u>This PDF file contains</u>:

- Supplementary Figures 1-10
- Supplementary Tables 1-4

a

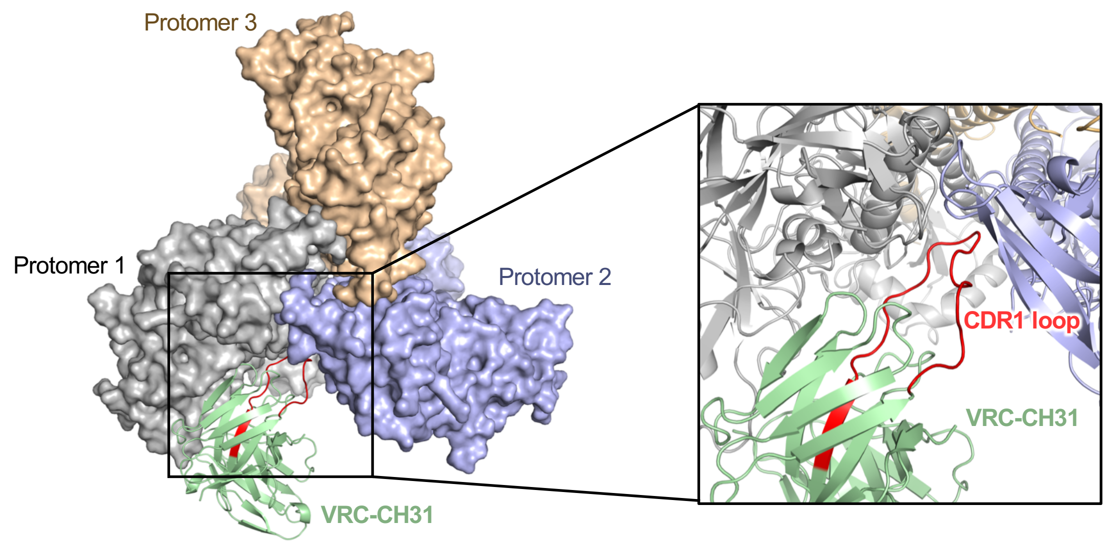


```
                   1         10        20        30                  40
                   |         |         |         |                   |
Heavy Chain        _____________FR1______________ ____CDR1_____ _______FR2_____
VH1-02*02          QVQLVQSGAEVKKPGASVKVSCKASGYTFT---------GYYMH WVRQAPGQGLEWMGWI
VRC-CH31           QVQLVQSGAAVRKPGASVTVSCKFAEDDDYSPYWVNPAPEHFIH FLRQAPGQQLEWLAWM
VRC01              QVQLVQSGGQMKKPGESMRISCRASGYEFI---------DCTLN WIRLAPGKRPEWMGWL
VRC07              QVRLSQSGGQMKKPGDSMRISCRASGYEFI---------NCPIN WIRLAPGKRPEWMGWM
VRC07-523-LS       QVRLSQSGGQMKKPGDSMRISCRASGYEFI---------NCPIN WIRLAPGKRPEWMGWM
N6                 RAHLVQSGTAMKKPGASVRVSCQTSGYTFT---------AHILF WFRQAPGRGLEWVGWI
3BNC117            QVQLLQSGAAVTKPGASVRVSCEASGYNIR---------DYFIH WWRQAPGQGLQWVGWI
VRC03              QVQLVQSGAVIKTPGSSVKISCRASGYNFR---------DYSIH WVRLIPDKGFEWIGWI
VRC06              EVQLVESGPVMRKPGSSMKISCATSGYNFR---------DFSIH WVRFNRRYGFEWIGWI
```

b

Neutralization potency (IC_{50}, μg/ml)

Virus	Clade	Tier	N6 WT	N6 CDR1-CH31	VRC07-523-LS WT	VRC07-523-LS CDR1-CH31
TRO11	B	2	0.204	0.246	0.128	0.185
25710	C	1B	0.158	0.533	0.105	0.686
398F1	A	2	0.108	0.144	0.034	0.032
CNE8	AE	2	0.123	0.306	0.084	0.094
X2278	B	2	0.062	0.100	0.008	0.023
BJOX002000	BC	2	0.045	>5	0.145	>5
X1632	G	2	0.029	0.030	0.012	0.012
CE1176	C	2	0.751	>5	0.293	0.695
246F3	AC	2	0.098	0.449	0.161	0.205
CH119	BC	2	0.074	>5	0.082	0.503
CE0217	C	2	0.078	0.305	0.056	0.127
CNE55	AE	2	0.079	0.062	0.018	0.034

Increased neutralization
- >3 fold
- 2-3 fold
- <2 fold

Decreased neutralization
- >3 fold
- 2-3 fold
- <2 fold

Supplementary Fig. 1 Engraftment of the elongated heavy-chain CDR1 loop of VRC-CH31 onto bNAbs N6 and VRC07-523-LS. **a** Upper panel, crystal structure of VRC-CH31 scFv complexed with BG505 SOSIP.664 (PDB ID: 6NNJ). The elongated CDR1 loop (red) of VRC-CH31 was shown to reach the adjacent gp120 protomer. Lower panel, sequence alignment shows the unique long CDR1 (red) of VRC-CH31 heavy chain. **b** Neutralization potency (IC_{50}) of two chimeric antibodies engrafted with the VRC-CH31 CDR1 loop, as evaluated against a small global panel of 12 HIV-1 strains. All neutralization assays were performed in duplicate wells.

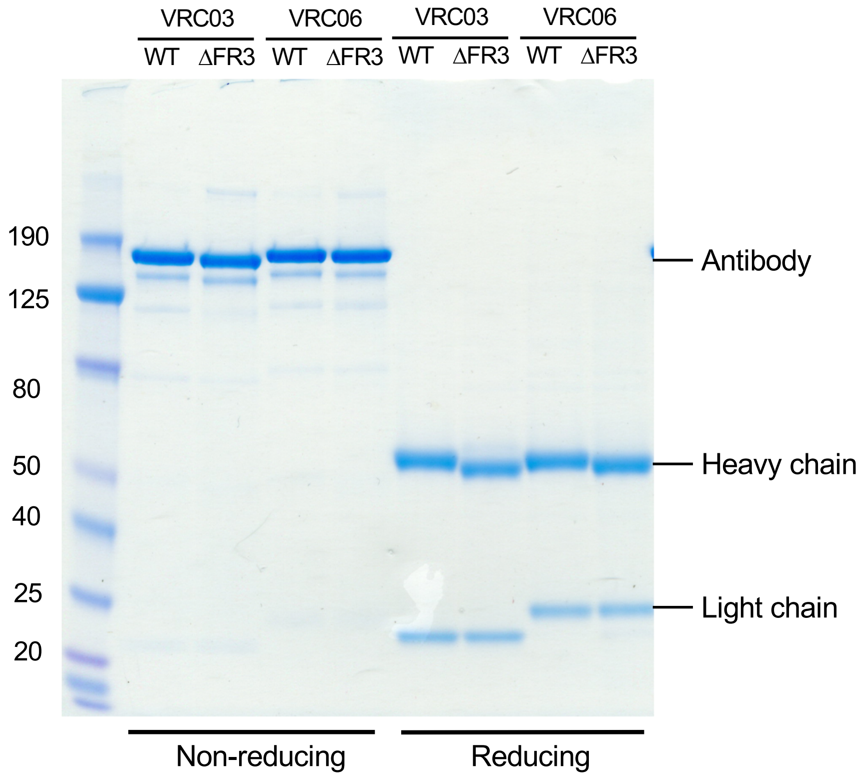

Supplementary Fig. 2 SDS-PAGE gel electrophoresis showing the correct molecular size of FR3-loop-deleted antibodies VRC03ΔFR3 and VRC06ΔFR3. The heavy chain of the chimeras has a lower molecular weight than the WT due to the FR3-loop deletion. The light chain is unchanged and identical to that of the WT.

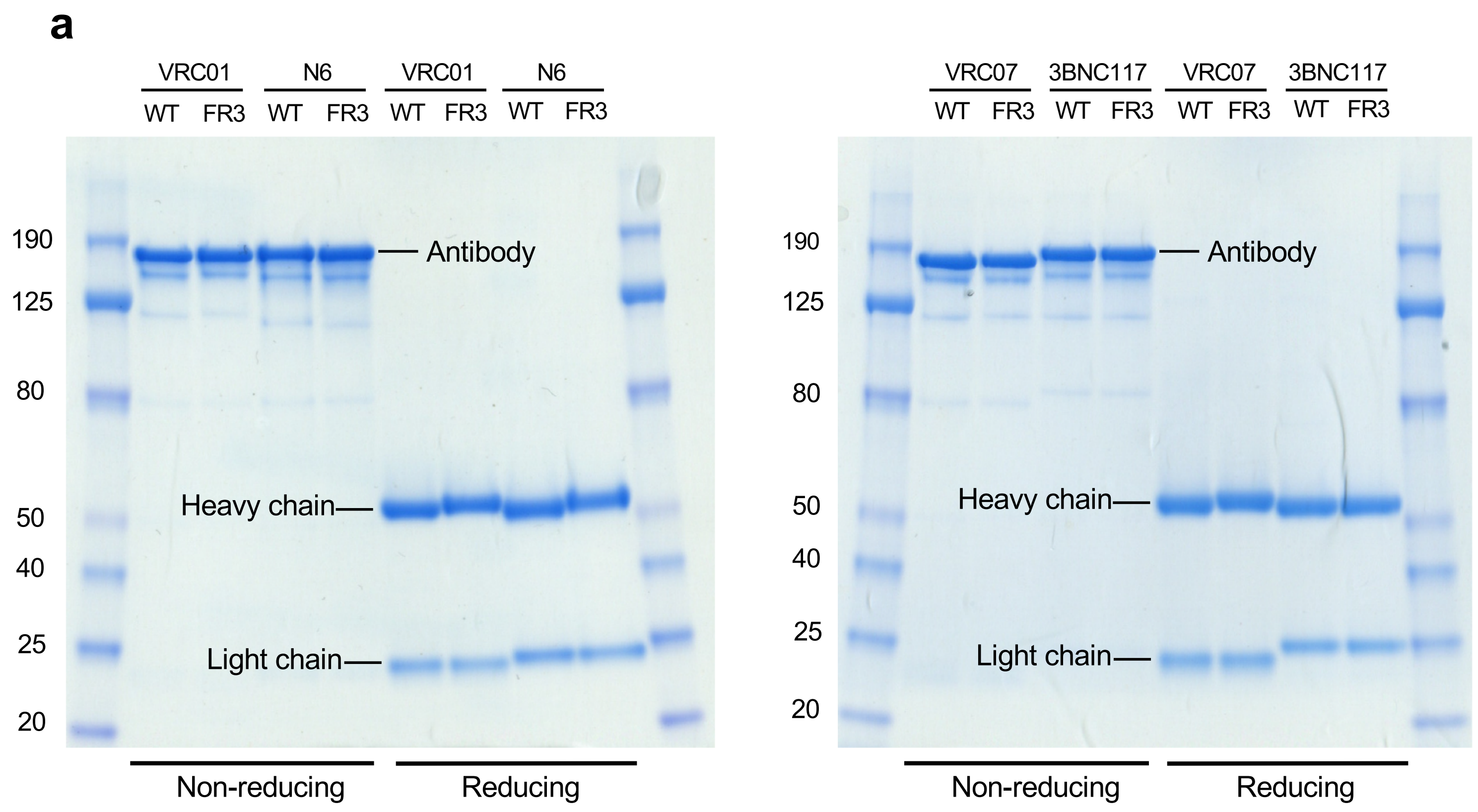

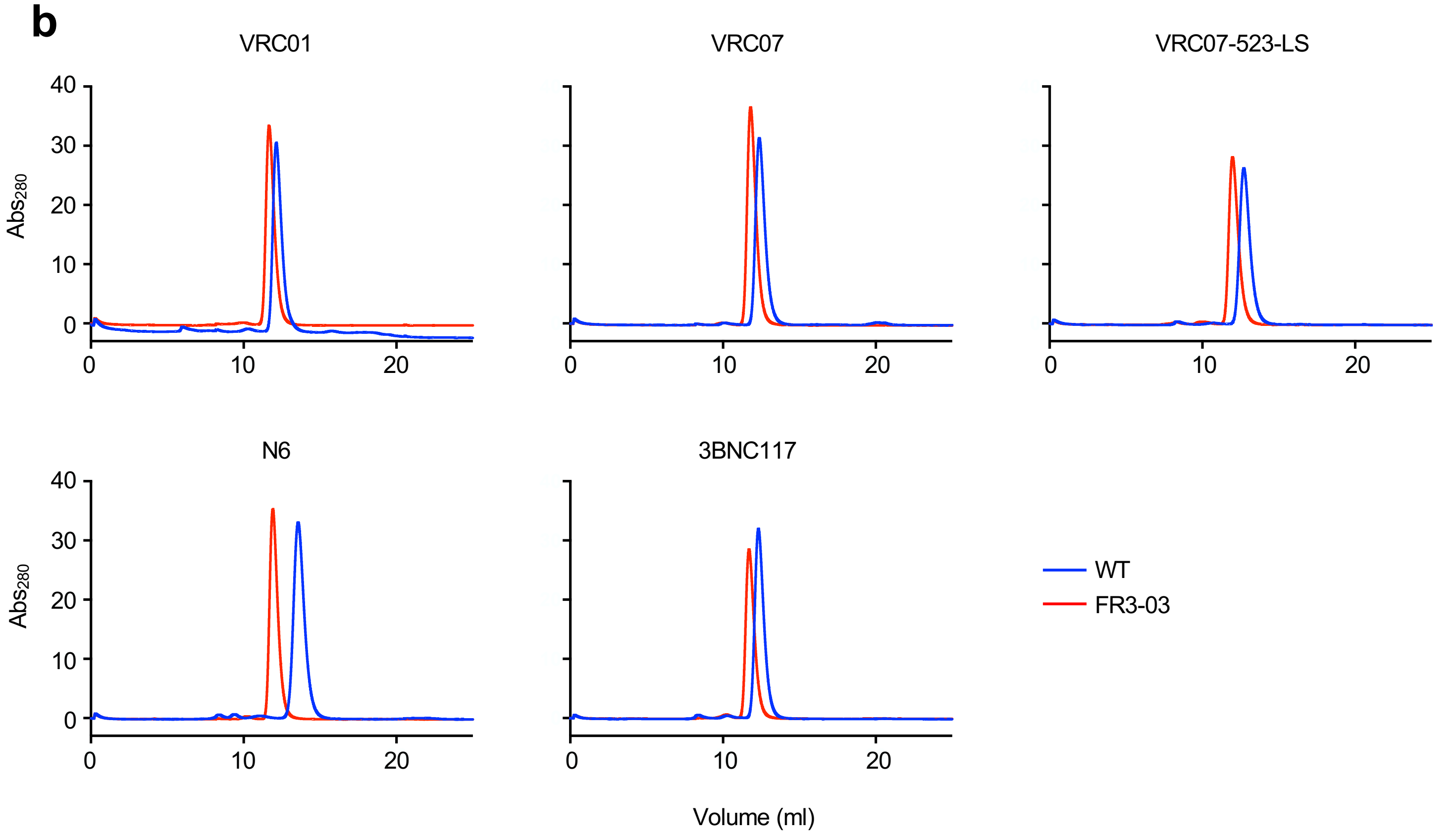

Supplementary Fig. 3 Molecular characterization of recombinant FR3-03 chimeric antibodies. **a** SDS-PAGE gel electrophoresis of WT and FR3-03 chimeric antibodies. The heavy chain of the chimeric antibody has a higher molecular weight due to FR3-03 engraftment, while the light chain is identical to that of the WT. **b** Size-exclusion chromatography profile of WT and FR3-03 chimeric antibodies.

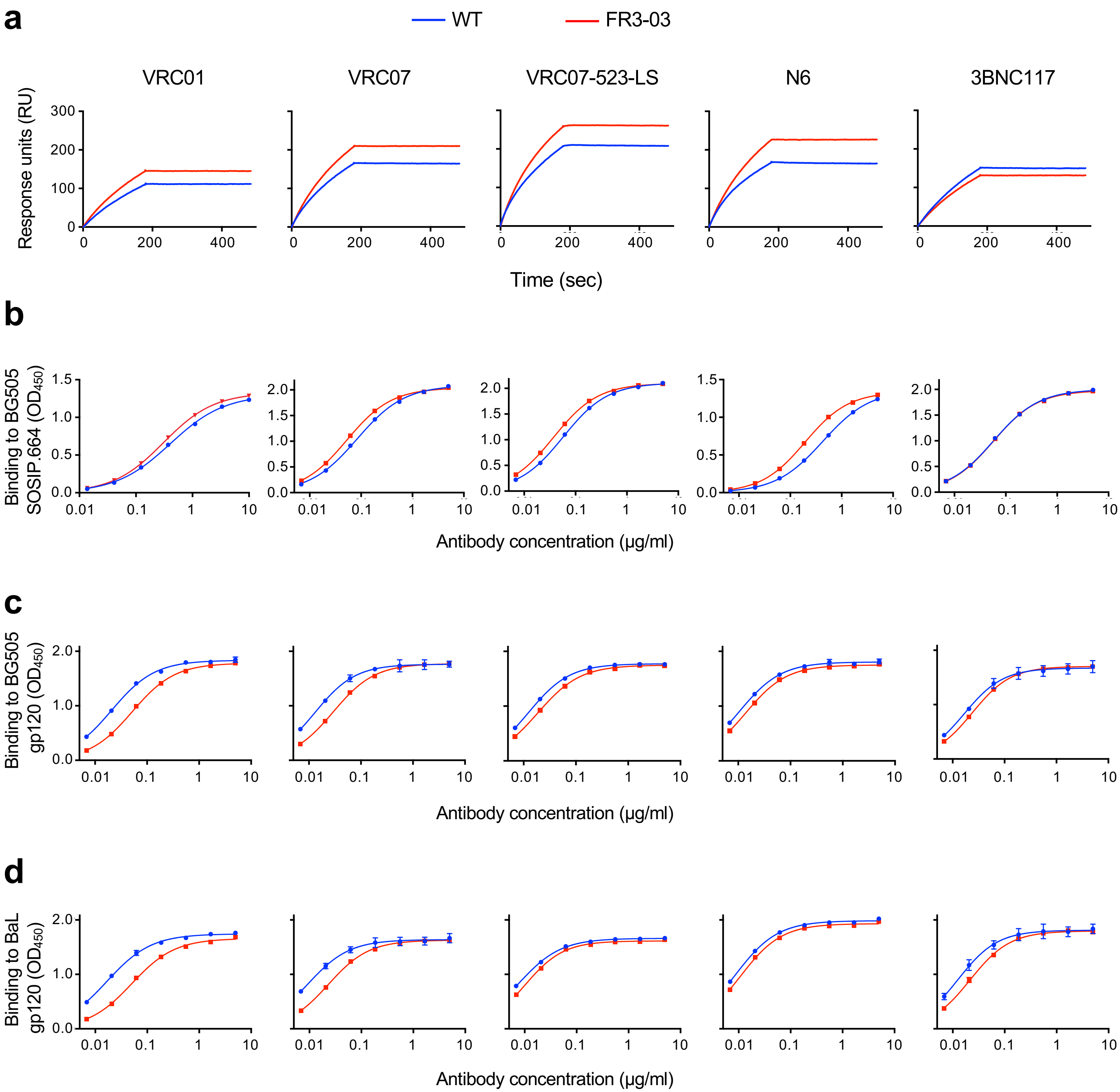

Supplementary Fig. 4 Binding of WT and FR3-chimeric antibodies to BG505 SOSIP.664 soluble trimers, as assessed by SPR (**a**) and ELISA (**b**). Antibody binding to monomeric gp120 derived from isolates BG505 (**c**) and BaL (**d**) was also tested by ELISA. For ELISA, data represent the mean (± SD) of two replicate wells from one representative experiment out of three independent experiments that were performed, which yielded similar results.

Neutralization potency (IC_{50}, µg/ml)

Virus	Clade	Tier	VRC01		VRC07		VRC07-523-LS		N6	
			WT	FR3-BNC	WT	FR3-BNC	WT	FR3-BNC	WT	FR3-BNC
TRO11	B	2	0.223	0.044	0.224	0.251	0.104	0.107	0.230	0.200
25710	C	1B	0.238	0.528	0.619	0.481	0.094	0.172	0.101	0.229
398F1	A	2	0.127	0.217	0.156	0.294	0.051	0.058	0.060	0.063
CNE8	AE	2	0.167	0.102	0.177	0.185	0.079	0.099	0.048	0.044
X2278	B	2	0.032	0.038	0.071	0.011	0.012	0.020	0.032	0.042
BJOX002000	BC	2	>5	4.588	>5	>5	0.082	2.227	0.087	0.317
X1632	G	2	0.044	0.064	0.037	0.032	0.013	0.015	0.062	0.029
CE1176	C	2	3.648	1.238	0.361	0.438	0.190	0.146	0.424	0.673
246F3	AC	2	0.361	0.147	0.444	0.491	0.143	0.199	0.082	0.178
CH119	BC	2	0.628	0.733	0.239	0.498	0.080	0.128	0.068	0.092
CE0217	C	2	0.070	0.065	0.099	0.098	0.040	0.043	0.036	0.069
CNE55	AE	2	0.025	0.077	0.183	0.073	0.028	0.057	0.054	0.050

Increased neutralization
- >3 fold
- 2-3 fold
- <2 fold

Decreased neutralization
- >3 fold
- 2-3 fold
- <2 fold

Supplementary Fig. 5 Neutralizing capacity (IC_{50}) of 3BNC117 FR3-chimeric antibodies against a small global panel of 12 HIV-1 strains. All neutralization assays were performed in duplicate wells.

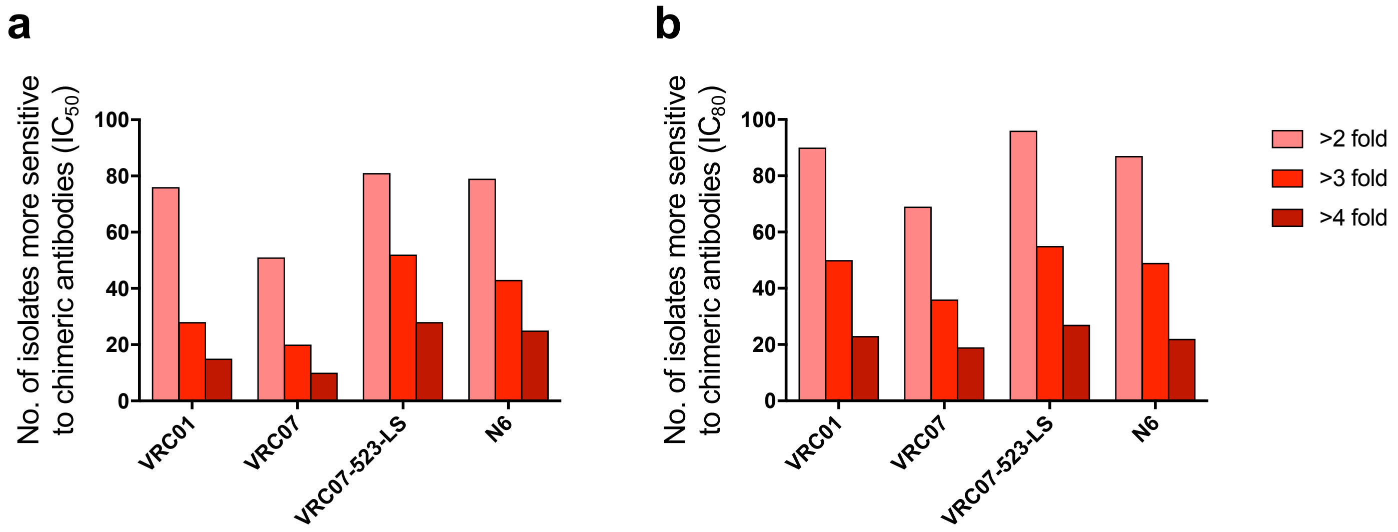

Supplementary Fig. 6 Increase in neutralizing capacity of chimeric antibodies against 208 HIV-1 global strains. The number of isolates that were more than 2-, 3- or 4-fold more sensitive to chimeric antibodies than to their respective original forms are plotted. **a** IC_{50} fold increases. **b** IC_{80} fold increases.

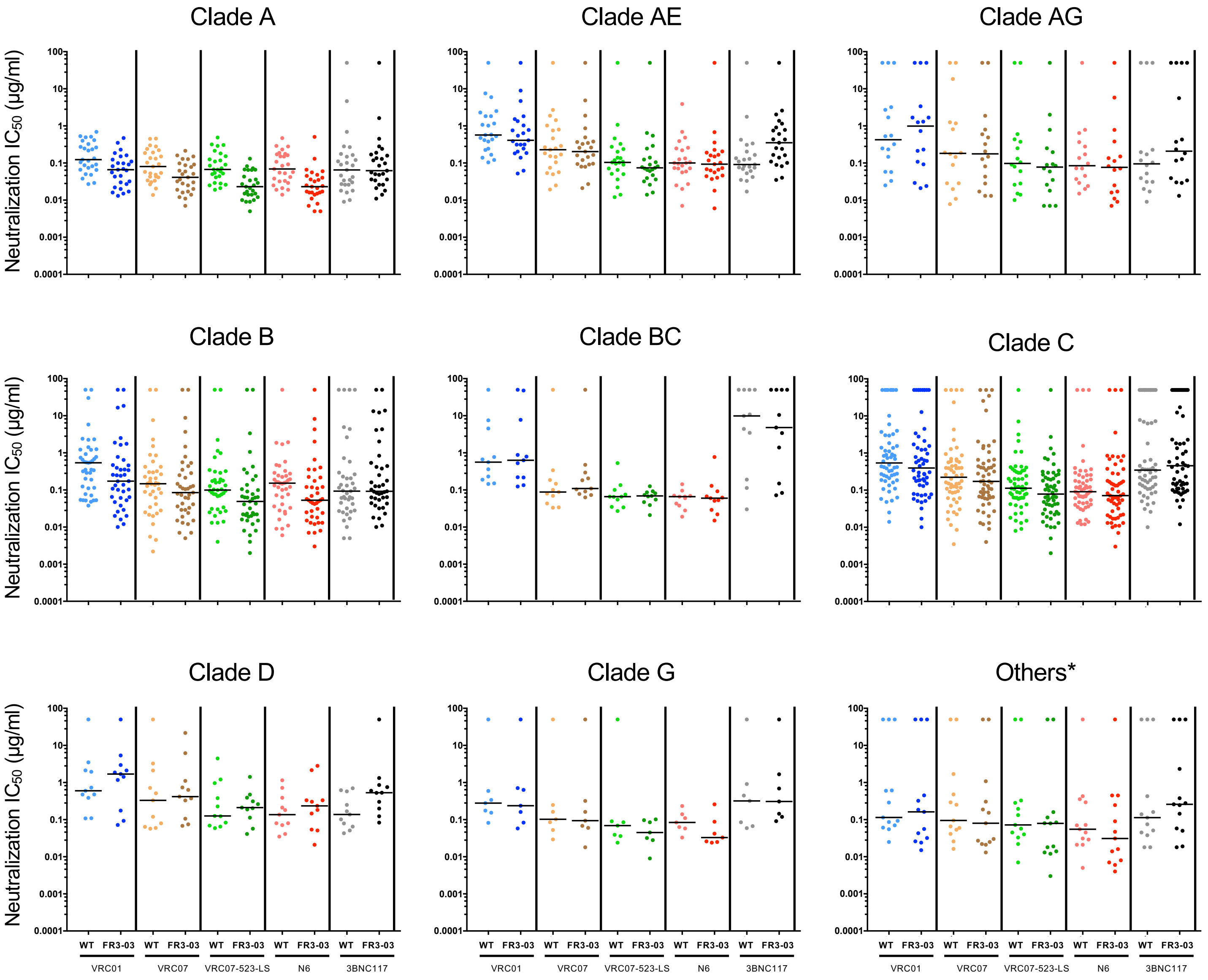

*Includes circulating recombinant forms (CRF) AC, ACD, AD and CD.

Supplementary Fig. 7 Neutralization potency of WT and FR3-chimeric antibodies on 208 global HIV-1 strains grouped by clade. The solid lines denote the median IC_{50} values (including neutralization-resistant strains). All neutralization assays were performed in duplicate wells.

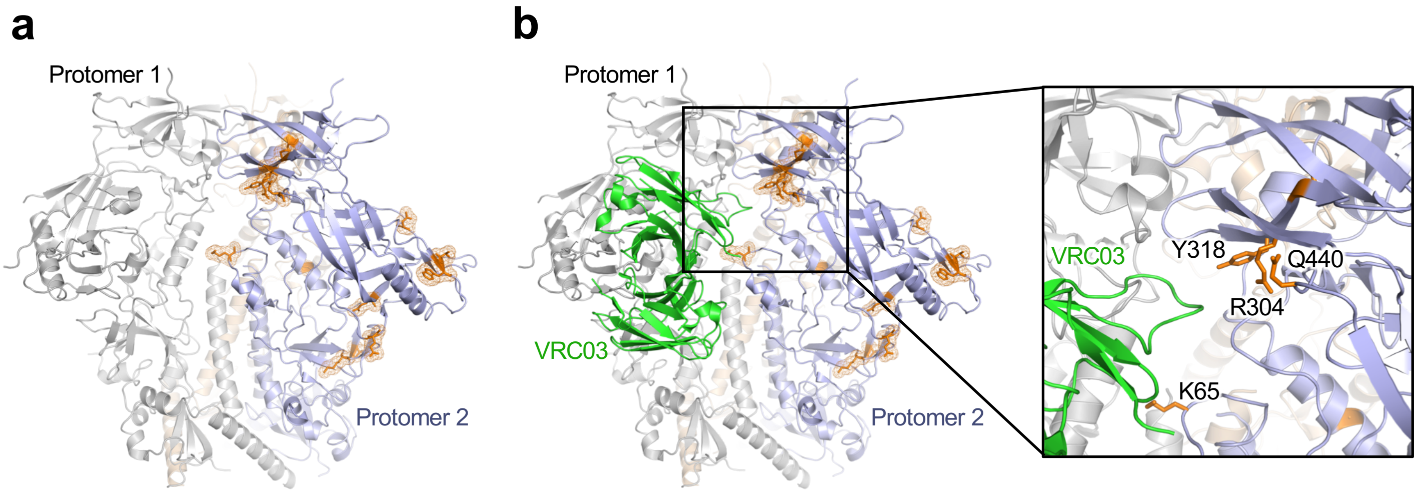

Supplementary Fig. 8 Amino acid positions associated with 1.5-fold or greater sensitivity change to FR3-chimeric antibodies in the 208 HIV-1 global Env panel, as identified by sequence analysis. Only positions common to more than one chimeric antibody and ranked among the top 20 are highlighted. **a** Amino acid positions associated with increased or decreased neutralization by 1.5 fold or greater are highlighted in orange on the structure of the BG505-SOSIP.664 trimer (PDB: 6CDI). **b** The same structure as in **a** bound to VRC03. The extended FR3 loop is shown in the close-up insert, highlighting the putative contact residues.

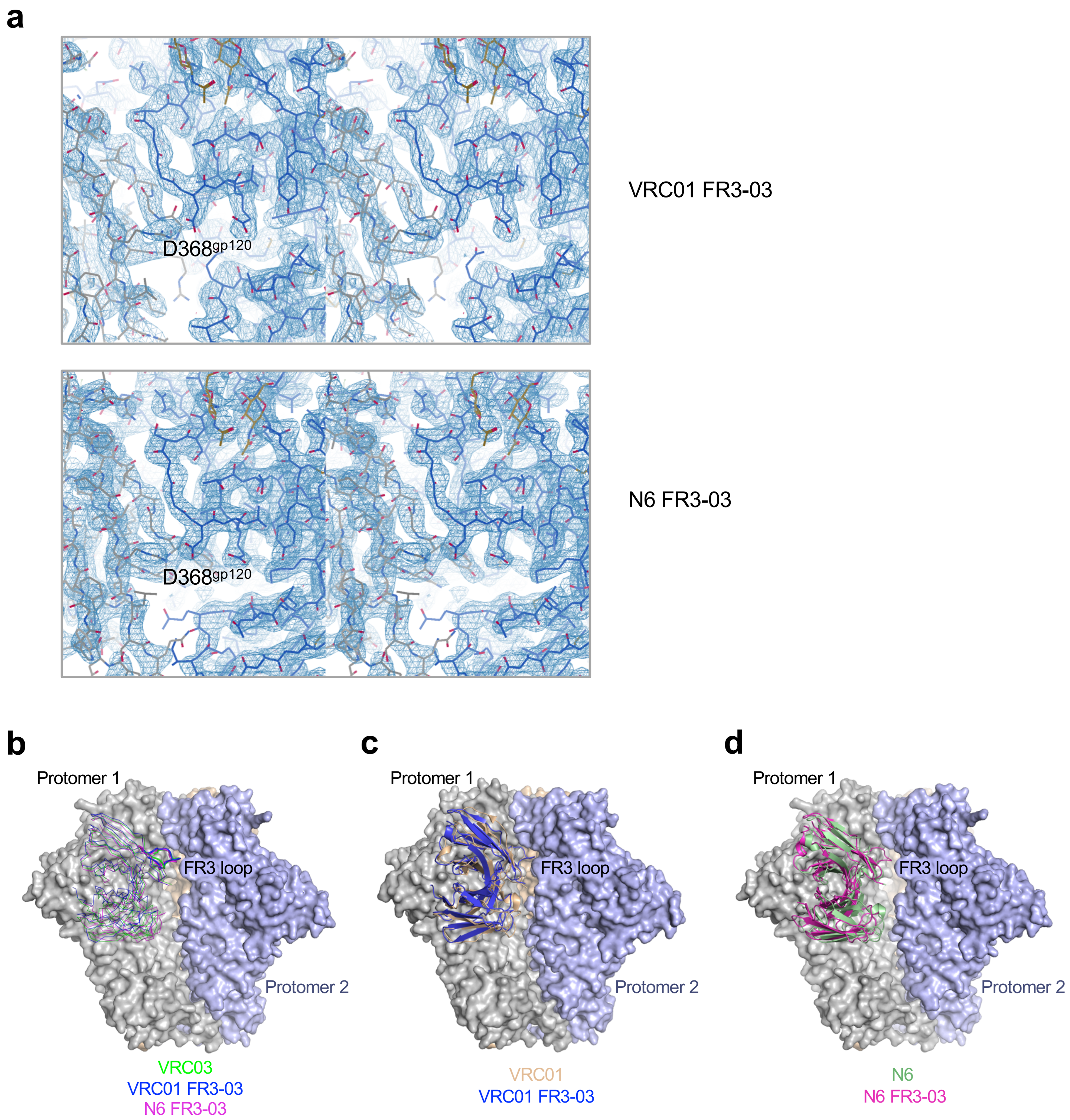

Supplementary Fig. 9 Stereo images of two crystal structures and structure alignments showing similar conformations and orientations of WT and FR3-chimeric antibodies in complex with soluble Env trimers. **a** Stereo images of two chimeric antibody structures (VRC01 FR3-03, PDB ID 6NNF; and N6 FR3-03, PDB ID 6NM6) in complex with a soluble trimer. 2Fo-Fc maps in blue meshes, centered around gp120 residue 368, are shown at 1.5σ contour level. **b** Alignment of BG505 SOSIP.664 trimers in complex with VRC01 FR3-03 (PDB ID: 6NNF), N6 FR3-03 (PDB ID: 6NM6) and VRC03 (PDB ID: 6CDI). The FR3 loop of and chimeric VRC01 (blue), N6 (magenta) and VRC03 (green) are highlighted. **c** Alignment between the JRFL SOSIP.664 trimer in complex with WT VRC01 (PDB ID: 5FYK) and the BG505 SOSIP.664 in complex with VRC01 FR3-03. **d** Alignment between gp120 in complex with WT N6 (PDB ID: 5TE6) and the BG505 SOSIP.664 trimer in complex with N6 FR3-03. The CH1 and CL domains of N6 were removed for clarity.

a

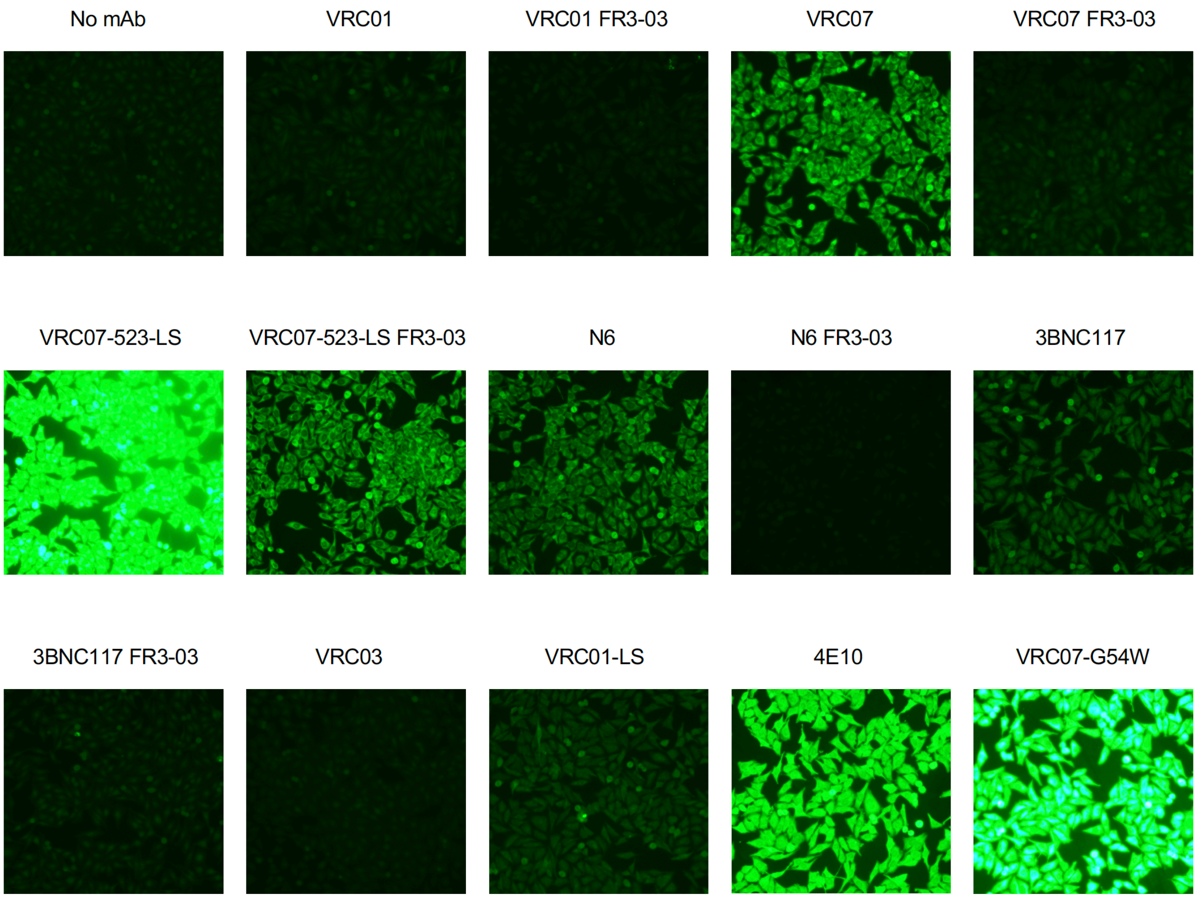

b

	OD at 450 nm		GPL units	
	**100 µg/ml**	**33.3 µg/ml**	**100 µg/ml**	**33.3 µg/ml**
VRC01	0.04	0.04	0.00	0.00
VRC01 FR3-03	0.03	0.03	0.00	0.00
VRC07	0.07	0.04	0.00	0.00
VRC07 FR3-03	0.04	0.03	0.00	0.00
VRC07-523-LS	0.25	0.11	0.00	0.00
VRC07-523-LS FR3-03	0.06	0.04	0.00	0.00
N6	0.17	0.08	3.32	0.00
N6 FR3-03	0.04	0.03	0.00	0.00
3BNC117	0.12	0.06	0.00	0.00
3BNC117 FR3-03	0.05	0.04	0.00	0.00
VRC03	0.04	0.04	0.00	0.00
VRC01-LS	0.06	0.04	0.00	0.00
4E10	2.33	2.40	173.01	178.20
VRC07-G54W	0.96	0.45	65.07	25.35

GPL score	Reactivity
< 20	Not reactive
20-80	Mildy reactive
> 80	Autoreactive

Supplementary Fig. 10 Autoreactivity of FR3-03 chimeric antibodies. **a** Autoreactivity of the WT and chimeric antibodies as assessed by immunofluorescence staining of Hep-2 epithelial cells. Antibodies were used at a concentration of 25 µg/mL. VRC01-LS, 4E10, VRC07-523-LS and VRC07-G54W served as controls. The data shown were from a representative experiment out of two performed which yielded similar results. **b** Antibody binding to cardiolipin as tested by ELISA. Control antibodies were the same as in **a**. All the autoreactivity results are summarized in Table 1.

Supplementary Table 1 Data collection and refinement statistics (molecular replacement) for the crystal structures of VRC01 FR3-03 (PDB ID: 6NNF), N6 FR3-03 (PDB ID: 6NM6), and VRC-CH31 (PDB ID: 6NNJ) in complex with the BG505 SOSIP.664 trimer.

	N6 FR3-03 scFv + BG505 SOSIP (with 3H109L Fab and 35O22 scFv)	VRC01 FR3-03 scFv + BG505 SOSIP (with 3H109L Fab and 35O22 scFv)	CH31 scFv + BG505 SOSIP (with 3H109L Fab and 35O22 scFv)
**Data collection**			
Space group	$P6_3$	$P6_3$	$P6_3$
Cell dimensions			
a, *b*, *c* (Å)	128.2, 128.2, 315.8	130.9, 130.9, 315.2	132.2, 132.2, 316.0
α, β, γ (°)	90, 90, 120	90, 90, 120	90, 90, 120
Resolution (Å)	50.0-3.2 (3.26-3.20); *50-2.7(2.80-2.75)*	50-3.5 (3.56-3.50); *50-2.7 (2.81-2.76)*	50-3.1 (3.15-3.10); *50-2.6 (2.64-2.60)*
R_{sym} or R_{merge} (%)	6.2 (44.0); *6.1 (53.4)*	5.2 (44.1); *5.3 (88.2)*	8.4 (66.1); *9 (252.3)*
$I / \sigma I$	12.2 (1.8); *12.4 (1.3)*	11.1 (1.7); *11.0 (0.9)*	17.3 (1.7); *17.6 (0.8)*
Completeness (%)	92.5 (56.2); *54.2 (2.1)*	87.0 (54.1); *40.7 (1.0)*	93.7 (54.7); *58.0 (0.5)*
Redundancy	2.8 (2.5); *2.9 (2.0)*	2.2 (2.0); *2.2 (1.8)*	5.9 (4.9); *6.2 (5.8)*
**Refinement**			
Resolution (Å)	37.0-2.7	42.8 - 2.7	43.3 - 2.6
No. reflections	34,666	27, 148	39,827
R_{work} / R_{free} (%)	23.6 / 28.6	24.0 / 29.7	25.0 / 29.0
No. atoms	11,906	12,009	11,963
Protein	11,299	11,347	11,384
Ligand/ion	607	662	579
Water	0	0	0
B-factors			
Protein	60.8	70.9	49.3
Ligand/ion	87.6	101.0	66.1
Water	n/a	n/a	n/a
R.m.s. deviations			
Bond lengths (Å)	0.003	0.003	0.005
Bond angles (°)	0.6	0.7	1.0

* Data processing statistics based on the overall resolution cutoff determined as: completeness greater than 50% and I/σI greater than 2, as mentioned in the Methods.

[#] Statistics for the highest-resolution shell are shown in parentheses.

[&] The diffraction limits were determined by the UCLA anisotropy server. Anisotropic data statistics are shown in italics.

Supplementary Table 2 Neutralization potency of FR3-chimeric antibodies against different Env mutants (IC50 fold-change relative to the WT virus). All neutralization assays were performed in duplicate wells.

	VRC01		VRC07		VRC07-523-LS		N6	
**Virus**	**WT**	**FR3-03**	**WT**	**FR3-03**	**WT**	**FR3-03**	**WT**	**FR3-03**
Wild-type	1.0	1.0	1.0	1.0	1.0	1.0	1.0	1.0
H66A	0.3	0.9	1.2	1.4	0.9	0.8	0.4	1.0
K207A	0.7	2.1	0.7	1.3	0.5	1.8	0.6	2.5
R304A	1.6	2.4	1.3	1.7	0.7	1.8	0.9	5.5
R304E	0.9	5.7	0.8	5.4	0.3	3.3	0.5	7.8
R308A	2.2	1.9	0.9	1.4	0.6	0.9	1.2	2.5
R308E	3.1	2.6	0.8	2.1	0.6	1.5	0.9	4.6
Y318A	2.0	1.3	1.2	1.4	0.8	1.1	0.7	2.3
Y318F	0.9	2.0	1.4	1.2	0.7	0.5	0.8	2.2
Y318E	0.6	5.6	0.2	5.0	0.5	4.1	0.8	9.0
R304A/R308A	0.6	2.2	0.7	2.2	0.5	2.6	0.7	8.4

Supplementary Table 3 NGS data sets derived from Short Read Archive.

PRJNA272713 Primer: VH1		PRJNA336331 Primer: 5'RACE		PRJNA209999 Primer: 5'RACE	
SRA:	Reads	SRA:	Reads	SRA:	Reads
SRR1767418	242415	SRR5471264	21706	SRR5471264	80504
SRR1767446	356702	SRR5471275	127003	SRR5471275	175274
SRR1767444	143887	SRR5471286	94289	SRR5471286	401985
SRR1767442	297053				
SRR1767441	293973				
SRR1767440	330783				
SRR1767438	2060				
SRR1767437	115290				
SRR1767436	3737				
SRR1767434	183357				
SRR1767432	163908				
SRR1767430	416799				
SRR1767429	377460				
SRR1767427	97679				
SRR1767425	117225				
SRR1767424	126556				
SRR1767423	111459				
SRR1767422	72737				
SRR1767421	205887				
SRR1767419	282292				
SRR1767418	242415				
Total Reads:	4183674	Total Reads:	242998	Total Reads:	657763

Supplementary Table 4 Sequence of DNA primers used in this study.

**VRC03H-del-F**	GAGTCTCTATGACTCGACAATTAGGCCCCGGCTGGGGCGTTGCC
**VRC03H-del-R**	GGCAACGCCCCAGCCGGGGCCTAATTGTCGAGTCATAGAGACTC
**VRC06H-del-F**	GCGTCTCAATGAGTCGTCTCTTTGGCCCCGGCACAGCCTAC
**VRC06H-del-R**	GTAGGCTGTGCCGGGGCCAAAGAGACGACTCATTGAGACGC
**VRC01-70-VRC03-F**	GACGACCCGGACTGGGGCACAGCCTTTTTGGAGCTG
**VRC01-70-VRC03-R**	TGGGTCTTGAGATAATTGTCGAGTCATGGTCACTCTG
**VRC01-70-VRC06-F**	TATTATCCGGACAGGGGCACAGCCTTTTTGGAGCTG
**VRC01-70-VRC06-R**	TAGGTCCTGAGAAAAGAGTCGAGTCATGGTCACTCTG
**VRC07-70-03-F**	GACGACCCGGACTGGGGCACAGCCTTTTTGGAGCTC
**VRC07-70-03-R**	TGGGTCTTGAGATAATTGTCGAGTCATGGTCACTCTG
**3BNC-70-03-F**	GACGACCCGGACTGGGGCTCCTTTTACATGGACCTG
**3BNC-70-03-R**	TGGGTCTTGAGATAATTGTCGAGTCAGACTGACTCTAC
**N6-70-03-F**	GACGACCCGGACTGGGGCATTGCGTACATGGACATC
**N6-70-03-R**	TGGGTCTTGAGATAATTGTCGAGTCAATGTGACCCTG
**VRC07-523-LS 70-03-F**	GACGACCCGGACTGGGGCACAGCCTTTTTGGAGCTC
**VRC07-523-LS 70-03-R**	TGGGTCTTGAGATAATTGTCGAGTCATGGTCACTCTG
**N6-70-BNC-F**	TGACACATTTTCCTTTTACATGGACATCAGAGGC
**N6-70-BNC-R**	AAGTCCCACGACGCGTGTCGAGTCAATGTGACCCTG
**VRC01-70-BNC-F**	TGACACATTTTCCTTTTTTTTGGAGCTGCGCTCG
**VRC01-70-BNC-R**	AAGTCCCACGACGCGTGTCGAGTCATGGTCACTCTG
**VRC07-70-BNC-F**	TGACACATTTTCCTTTTTTTTGGAGCTCCGTTCC
**VRC07-70-BNC-R**	AAGTCCCACGACGCGTGTCGAGTCATGGTCACTCTG
**VRC07-CH31-F**	CCATACTGGGTGAATCCGGCCCCTGAATGTCCAATAAATTGGATTCG
**VRC07-CH31-R**	AGAGTAGTCGTCGTCTTCAGCGAATTTACAAGAAATTCTCATCGAG
**N6-CDR1-CH31-F**	GTGAATCCGGCCCCTGAACATTTTATTCACTTTTTGCGACAGGCCCCCGGGCGA
**N6-CDR1-CH31-R**	CCAGTATGGAGAGTAGTCGTCGTCTTCAGCGAATTTGCAGGAGACTCTTACTGAGGCCCCC
