# Supplementary material for: Improvement of antibody functionality by structure-guided paratope engraftment
Source: Nat Commun. 2019 Feb 13;10:721. doi: 10.1038/s41467-019-08658-4 (PMC6374468; doi:10.1038/s41467-019-08658-4)
Supplement: Supplementary file 3 — Description of Additional Supplementary Files [file 41467_2019_8658_MOESM3_ESM.docx]

**Description of Additional Supplementary Files**

File Name: Supplementary Datasets 1

Description: Sensitivity (IC50) of a large global HIV-1 Env panel to VRC03-FR3-loop chimeric bNAbs.

File Name: Supplementary Dataset 2

Description: Sensitivity (IC80) of a large global HIV-1 Env panel to VRC03-FR3-loop chimeric bNAbs.

File Name: Supplementary Dataset 3

Description: Association of neutralization sensitivity with aa. sequence signatures in 208 HIV-1 Envs.
