# Supplementary material for: Improvement of antibody functionality by structure-guided paratope engraftment
Source: Nat Commun. 2019 Feb 13;10:721. doi: 10.1038/s41467-019-08658-4 (PMC6374468; doi:10.1038/s41467-019-08658-4)
Supplement: Supplementary file 7 — Reporting Summary [file 41467_2019_8658_MOESM7_ESM.pdf]

natureresearch

Corresponding author(s): Paolo Lusso

Reporting Summary

Nature Research wishes to improve the reproducibility of the work that we publish. This form provides structure for consistency and transparency in reporting. For further information on Nature Research policies, see Authors & Referees and the Editorial Policy Checklist.

Statistical parameters

When statistical analyses are reported, confirm that the following items are present in the relevant location (e.g. figure legend, table legend, main text, or Methods section).

n/a	Confirmed	
☐	☒	The exact sample size (*n*) for each experimental group/condition, given as a discrete number and unit of measurement
☐	☒	An indication of whether measurements were taken from distinct samples or whether the same sample was measured repeatedly
☐	☒	The statistical test(s) used AND whether they are one- or two-sided<br>*Only common tests should be described solely by name; describe more complex techniques in the Methods section.*
☐	☒	A description of all covariates tested
☒	☐	A description of any assumptions or corrections, such as tests of normality and adjustment for multiple comparisons
☐	☒	A full description of the statistics including central tendency (e.g. means) or other basic estimates (e.g. regression coefficient) AND variation (e.g. standard deviation) or associated estimates of uncertainty (e.g. confidence intervals)
☐	☒	For null hypothesis testing, the test statistic (e.g. *F*, *t*, *r*) with confidence intervals, effect sizes, degrees of freedom and *P* value noted<br>*Give P values as exact values whenever suitable.*
☒	☐	For Bayesian analysis, information on the choice of priors and Markov chain Monte Carlo settings
☒	☐	For hierarchical and complex designs, identification of the appropriate level for tests and full reporting of outcomes
☒	☐	Estimates of effect sizes (e.g. Cohen's *d*, Pearson's *r*), indicating how they were calculated
☐	☒	Clearly defined error bars<br>*State explicitly what error bars represent (e.g. SD, SE, CI)*

Our web collection on statistics for biologists may be useful.

Software and code

Policy information about availability of computer code

Data collection	Biacore 3000 Control Software 3.2
Data analysis	GraphPad Prism 7, BIAevaluation software 4.1, IgBlast 1.8

For manuscripts utilizing custom algorithms or software that are central to the research but not yet described in published literature, software must be made available to editors/reviewers upon request. We strongly encourage code deposition in a community repository (e.g. GitHub). See the Nature Research guidelines for submitting code & software for further information.

Data

Policy information about availability of data

All manuscripts must include a data availability statement. This statement should provide the following information, where applicable:

- Accession codes, unique identifiers, or web links for publicly available datasets
- A list of figures that have associated raw data
- A description of any restrictions on data availability

All data generated in this study are available within this paper and the supplementary files.

Field-specific reporting

Please select the best fit for your research. If you are not sure, read the appropriate sections before making your selection.

☒ Life sciences ☐ Behavioural & social sciences ☐ Ecological, evolutionary & environmental sciences

For a reference copy of the document with all sections, see nature.com/authors/policies/ReportingSummary-flat.pdf

Life sciences study design

All studies must disclose on these points even when the disclosure is negative.

Sample size	The sample size for pharmacokinetics studies was determined as to provide sufficient numbers of animals for a reliable comparison (N = 5 for mice and N = 3 for nonhuman primates.
Data exclusions	No data were excluded.
Replication	All replications were successful.
Randomization	For pharmacokinetics studies, the animals were allocated to groups randomly.
Blinding	Both animal handlers and investigators were blinded for pharmacokinetics studies, but not for in vitro assays performed with specific antibodies.

Reporting for specific materials, systems and methods

Materials & experimental systems

n/a	Involved in the study
☐	☒ Unique biological materials
☐	☒ Antibodies
☐	☒ Eukaryotic cell lines
☒	☐ Palaeontology
☐	☒ Animals and other organisms
☒	☐ Human research participants

Methods

n/a	Involved in the study
☒	☐ ChIP-seq
☒	☐ Flow cytometry
☒	☐ MRI-based neuroimaging

Unique biological materials

Policy information about availability of materials

Obtaining unique materials	There are no restrictions on availability of unique materials.

Antibodies

Antibodies used	The following antibodies were used: Human monoclonal antibodies VRC03, VRC03delFR3, VRC06, VRC06delFR3, VRC01, VRC01 FR3-03, VRC07, VRC07 FR3-03, VRC07-523-LS, VRC07-523-LS FR3-03, N6, N6 FR3-03, 3BNC117, 3BNC117 FR3-03, VRC01-LS, VRC07-G54W, 4E10, 35O22 and PGT122, and respective Fabs or scFvs produced and purified in-house; HRP-conjugated goat anti-human IgG (Cat# A8419) from Sigma, HRP-conjugated goat anti-human IgG (Cat# 109-035-008) from Jackson ImmunoResearch.
Validation	Commercial antibodies were tested and titrated before use. In-house produced antibodies were validated by SDS-PAGE under reducing and nonreducing conditions, UV absorbance measured by NanoDrop, and binding titration on HIV-1 Env-expressing cells.

Eukaryotic cell lines

Policy information about cell lines

Cell line source(s)	All cell lines were sourced from the ATCC collection or obtained from the VRC, NIH (Dr. Peter D. Kwong's and Dr. John R. Mascola's labs).
Authentication	Cell lines were validated against published standards.
Mycoplasma contamination	No mycoplasma contamination was detected.
Commonly misidentified lines (See ICLAC register)	No misidentified cell lines were used.

Animals and other organisms

Policy information about studies involving animals; ARRIVE guidelines recommended for reporting animal research

Laboratory animals	Rhesus macaques (Macaca mulatta), 3-6 year old, both male and female; human FcRn transgenic mice (strain: C57BL/6, B6.mFcRn−/− hFCRN Tg32), 4 weeks old, female.
Wild animals	No wild animals were used in this study.
Field-collected samples	No field-collected samples were used in this study.
