# Supplementary material for: Improvement of antibody functionality by structure-guided paratope engraftment
Source: Nat Commun. 2019 Feb 13;10:721. doi: 10.1038/s41467-019-08658-4 (PMC6374468; doi:10.1038/s41467-019-08658-4)
Supplement: Supplementary file 2 — Peer Review File [file 41467_2019_8658_MOESM2_ESM.pdf]

This manuscript has been previously reviewed at another journal that is not operating a transparent peer review scheme. This document only contains reviewer comments and rebuttal letters for versions considered at Nature Communications .

Reviewers' Comments:

Reviewer #1:
None

Reviewer #2:
Remarks to the Author:
The authors have adequately addressed the reviewer comments from the prior submission.
